# Ongoing Recombination in SARS-CoV-2 Revealed through Genealogical Reconstruction

**DOI:** 10.1093/molbev/msac028

**Published:** 2022-02-02

**Authors:** Anastasia Ignatieva, Jotun Hein, Paul A Jenkins

**Affiliations:** 1 Department of Statistics, University of Warwick, Coventry, United Kingdom; 2 Department of Statistics, University of Oxford, Oxford, United Kingdom; 3 The Alan Turing Institute, British Library, London, United Kingdom; 4 Department of Computer Science, University of Warwick, Coventry, United Kingdom

**Keywords:** SARS-CoV-2, genealogy, recombination, parsimony

## Abstract

The evolutionary process of genetic recombination has the potential to rapidly change the properties of a viral pathogen, and its presence is a crucial factor to consider in the development of treatments and vaccines. It can also significantly affect the results of phylogenetic analyses and the inference of evolutionary rates. The detection of recombination from samples of sequencing data is a very challenging problem and is further complicated for SARS-CoV-2 by its relatively slow accumulation of genetic diversity. The extent to which recombination is ongoing for SARS-CoV-2 is not yet resolved. To address this, we use a parsimony-based method to reconstruct possible genealogical histories for samples of SARS-CoV-2 sequences, which enables us to pinpoint specific recombination events that could have generated the data. We propose a statistical framework for disentangling the effects of recurrent mutation from recombination in the history of a sample, and hence provide a way of estimating the probability that ongoing recombination is present. We apply this to samples of sequencing data collected in England and South Africa and find evidence of ongoing recombination.

## Introduction

Ongoing mutation of the SARS-CoV-2 virus has received significant scientific and media attention since the start of the pandemic. The process of viral recombination has received far less coverage, but has the potential to have a drastic impact on the evolution of virulence, transmissibility, and evasion of host immunity (Simon-Loriere and Holmes 2011). Recombination occurs when host cells are coinfected with different strains of the same virus, and during replication the genomes are reshuffled and combined before being packaged and released as new offspring virions, now potentially possessing very different pathogenic properties. This makes the presence of recombination a crucial factor to consider when developing vaccines and treatments. Although the role of recombination between different coronaviruses in the emergence of SARS-CoV-2 has been widely studied, understanding its potential for ongoing recombination within human hosts has proved difficult.

The detection of ongoing recombination from a sample of genetic data is, in general, a very challenging problem. Only a fraction of recombination events significantly change the shape of a genealogy, and even then, mutations must occur on the correct branches of the genealogy in order to create patterns that are detectable in the data (Hein et al. 2004, Section 5.11). In evolutionary terms, a relatively short time period has passed since the start of the pandemic, so typical SARS-CoV-2 sequences differ only by a small number of mutations, meaning that recombination events are likely to be undetectable or leave only faint traces. Moreover, the effects of recombination can be indistinguishable from those of recurrent mutation (McVean et al. 2002), where mutations have occurred at the same site multiple times in the history of the sample; recurrent mutation is known to be a common feature of SARS-CoV-2 evolution (e.g., van Dorp, Acman, et al. 2020), and thus distinguishing between the effects of recurrent mutation and recombination is an important goal. Coronaviruses are known to have relatively high recombination rates (Su et al. 2016), and cell culture studies indicate that this holds true for SARS-CoV-2 (Gribble et al. 2021). This suggests that ongoing intra-host recombination since the start of the pandemic should be commonplace, but detection efforts are thwarted by the slow accumulation of genetic diversity.

Early evidence of ongoing recombination in SARS-CoV-2 was presented by Yi (2020), who identified the presence of loops in reconstructed phylogenetic networks, which can arise as a consequence of recombination. A number of more recent reports have utilized methods based on classifying sequences into clades, and searching for those that appear to carry a mix of mutations characteristic to more than one clade. VanInsberghe et al. (2021) identified 1,175 possible recombinants out of 537,000 analyzed sequences; Varabyou et al. (2021) identified 225 possible recombinants out of 88,000; Jackson et al. (2021) have identified a small number of putative recombinants circulating in the United Kingdom. These methods are sensitive to the classification of sequences into clades, do not allow for the detection of intra-clade recombinants (thus underestimating the overall extent of recombination), and do not incorporate a framework for quantifying how likely it is that an observed pattern of incompatibilities has arisen through recombination rather than recurrent mutation. A number of studies have also failed to detect recombination signal, through the analysis of linkage disequilibrium and similar techniques (De Maio et al. 2020; Nie et al. 2020; Richard et al. 2020; Tang et al. 2020; Wang et al. 2020; van Dorp, Richard, et al. 2020). In general, a relatively small number of putative recombinant sequences have been identified to date, and there is a lack of compelling evidence for widespread recombination in SARS-CoV-2. Given the aforementioned causes for studies to be underpowered, the overall extent and importance of ongoing recombination for SARS-CoV-2 remains not fully resolved.

Phylogenetic analysis of SARS-CoV-2 data largely assumes the absence of recombination. Recombination can significantly influence the accuracy of phylogenetic inference (Posada and Crandall 2002), distorting the branch lengths of inferred trees and making mutation rate heterogeneity appear stronger (Schierup and Hein 2000). Moreover, when analyzing data at the level of consensus sequences, the genealogy of a sample is related to the transmission network of the disease, with splits in the genealogy relating to the transmission of the virus between hosts. Models used for constructing genealogies and inferring evolutionary rates for this type of data cannot fully incorporate potentially important factors, such as geographical structure, patterns of social mixing, travel restrictions, and other nonpharmaceutical interventions, without making inference intractable. Relying on standard tree-based models can easily lead to biased estimates (Jensen and Kowalik 2020), with the extent of the error due to model misspecification being very difficult to quantify.

In this article, we use KwARG (Ignatieva et al. 2021), a parsimony-based method for reconstructing possible genealogical histories, to detect and examine crossover recombination events in samples of viral consensus sequences. This approach provides a concrete way of describing their genealogical relationships, sidestepping the challenges presented by discrepancies in clade assignment, enabling the detection of intraclade recombination, avoiding the need to specify a particular model of evolution, and allowing for the explicit identification of possible recombination events in the history of a sample. Our method naturally handles both recombination and recurrent mutation, identifying a range of possible explicit genealogical histories for the data set with varying proportions of both event types. Rather than using summary statistics calculated from the data, or focusing only on patterns of clade-defining SNPs, our method utilizes all of the information contained in the patterns of incompatibilities observed in a sample, allowing for powerful detection and identification of possible recombinants. Moreover, we provide a nonparametric framework for evaluating the probability of a given number of recurrent mutations, thus quantifying how many recombinations are likely to have occurred in the history of a data set. This allows for a more thorough and statistically principled assessment of the extent to which ongoing recombination is occurring.

We investigate the presence of ongoing recombination in SARS-CoV-2 using publicly available data from GISAID (Elbe and Buckland-Merrett 2017), collected between November 2020 and February 2021. Using data from South Africa, we demonstrate that our method can detect recombination both when the sample contains sequences from multiple distinct lineages (“interclade”), as well as all from the same lineage (“intraclade”). Further, we show that our method can accurately detect consensus sequences carrying patterns of mutations that are consistent with recombination, flagging these sequences for further investigation—and we demonstrate, using data from England, that it can identify both sequences arising as a result of sequencing errors due to sample contamination, aiding in identifying quality control issues, as well as sequences likely to be true recombinants. We validate our method using extensive simulation studies, and through application to MERS-CoV data, for which we find evidence of recombination, in agreement with previous studies.

## Results

### Overview of Methods

Our method consists of two main steps. First, using KwARG, plausible genealogical histories are reconstructed for each sample under a parsimony assumption, with varying proportions of posited recombination and recurrent mutation events. Then, using simulation, we approximate the distribution of the number of recurrent mutations that might be observed in a data set of the same size as each sample. We use this to establish which of the identified genealogical histories is more plausible for the data at hand, and thus whether the presence of recombination events in the history of the given sample is likely.

This can be framed in the language of statistical hypothesis testing. The “null hypothesis” is the absence of recombination. The test statistic *T* is the number of recurrent mutations in the history of the data set; the null distribution of *T* is approximated through simulation. The observed value *T*_obs_ is the minimal number of recurrent mutations required to explain the data set in the absence of recombination, as estimated by KwARG. The ”*P* value” is the probability of observing a number of recurrent mutations equal to or greater than *T*_obs_. Small *P* values allow us to reject the null hypothesis, providing evidence that recombination has occurred. The reconstructed genealogies then allow for the detailed examination of possible recombination events in the history of the sampled sequences.

We emphasize that we make very conservative assumptions throughout, both in processing the data and in estimating the distribution of the number of recurrent mutations. Moreover, the number of recurrent mutations required to explain a given data set computed by KwARG is (or is close to) a lower bound on the actual number of such events, and is likely to be an underestimate, making the reported *P* values larger (more stringent).

#### Reconstruction of Genealogies

All of the viral particles now in circulation had a common ancestor at the time of emergence of the virus, so sequences sampled at the present time can be united by a network of evolution going back to this shared ancestor through shared predecessors, termed the *ancestral recombination graph* (*ARG*) (Griffiths and Marjoram 1997). As the sample consists of consensus sequences (at the level of one sequence per host), an edge of this network represents a viral lineage, possibly passing through multiple hosts before being sequenced at the present. An example of an ARG topology can be seen in figure 3. Mutations are represented as points on the edges, labeled by the sites they affect. Considering the graph backwards in time (from the bottom up), the point at which two edges merge represents the time at which some sequences in the data coalesced, or have found a common ancestor. A point at which an edge splits into two corresponds to a recombination—the parts of the genome to the left and to the right of the breakpoint (whose site number is labeled inside the blue recombination node) are inherited from two different parent particles. The network thus fully encodes the evolutionary events in the history of a sample.

Both recombination and recurrent mutation can produce patterns of *incompatible* sites in the data, which violate the four gamete test (Hudson and Kaplan 1985) and could not have been generated through replication and nonhomoplasic mutations alone. KwARG reconstructs possible ARGs for a given data set under the *parsimony* assumption, seeking to minimize the posited number of recombination and/or recurrent mutation events. Our method requires the computation of a *lower bound* on the number of events in the evolutionary history (we are, in essence, deliberately seeking a biased estimate, to make our detection probabilities more conservative). Crucially, the parsimony approach does this without requiring the assumption of a particular generative model for the data (such as the coalescent with exponential growth) beyond specifying the types of events that can occur. Although this means that mutation and recombination *rates* cannot be inferred, it allows us to sidestep the need to specify a detailed model of population dynamics, which is particularly challenging for SARS-CoV-2 data. A parsimony-based approach is more appropriate when our focus is on interrogating the hypothesis that recombination is present at all. It also allows for the explicit reconstruction of possible events in the history of a sample, and thus allows us to identify recombinant sequences and uncover patterns consistent with the effects of sequencing errors.

KwARG outputs a set of possible ARGs for a given data set, including those that explain all incompatibilities through recombination events, those that only contain recurrent mutations, and those containing a combination of both event types. KwARG distinguishes between recurrent mutations that occur on the internal branches of the ARG from those can be placed on the terminal branches, which affect only one sequence in the input data set, so can be examined separately for indications that they arose due to errors in the sequencing process.

We ran KwARG on four samples of data: from South Africa, collected in 1) November 2020 (50 sequences, with 25 from lineage B.1.351, and 25 from other lineages) and 2) February 2021 (38 sequences, all from lineage B.1.351), and from England, collected in 3) November 2020 (80 sequences, with 40 sequences from lineage B.1.1.7 and 40 from other lineages within GISAID clade GR), and 4) December 2020–January 2021 (40 sequences within GISAID clade GR). An overview of the identified solutions is given in table 1*a–d*.

**Table 1.** Summary of Solutions Identified by KwARG for Each Sample, and the Probability of Observing the Corresponding Number of Recurrent Mutations.

*R*	*RM*	P(RM)	*P*
(*a*) South Africa (Nov.)			
10	0	0.28	1.00
8	1	0.35	0.72
6	2	0.23	0.37
4	3	0.10	0.14
3	4	0.03	0.04
2	5	0.01	0.01
1	7	0.00	$4\times 10^{-4}$
0	9	0.00	$7\times 10^{-6}$
(*b*) South Africa (Feb.)			
7	0	0.52	1.00
5	1	0.34	0.48
3	2	0.11	0.14
2	3	0.03	0.03
1	4	0.00	$5\times 10^{-3}$
0	5	0.00	$7\times 10^{-4}$
(*c*) England (Jan.)			
10	0	0.11	1.00
8	1	0.24	0.89
6	2	0.27	0.65
4	3	0.20	0.38
3	4	0.11	0.19
2	5	0.05	0.08
1	6	0.02	0.03
0	14	0.00	$1\times 10^{-6}$
(*d*) MERS-CoV			
9	0	0.42	1.00
7	1	0.36	0.58
6	2	0.16	0.22
5	3	0.05	0.06
4	4	0.01	0.01
3	5	0.00	$2\times 10^{-3}$
2	10	0.00	$<1\times 10^{-6}$
1	12	0.00	$<1\times 10^{-6}$
0	16	0.00	$<1\times 10^{-6}$

Note.—First column: number of recombinations. Second column: number of recurrent mutations. Third column: probability of observing a number of recurrent mutations equal to that in the second column. Fourth column: corresponding *P* values (probability of observing a number of recurrent mutations equal to or greater than that in the second column).

#### Evaluation of Solutions

We next determined which of the solutions identified by KwARG is more likely, by calculating the probability of observing the given number of recurrent mutations. To avoid making model-based assumptions on the genealogy of the sample, we use a nonparametric method inspired by the *homoplasy test* of Maynard Smith and Smith (1998), which estimates the probability of observing the minimal number of recurrent mutations required to generate the sample in the absence of recombination (i.e., if the shape of the genealogy is constrained to be a tree). If this probability is very small, then it provides evidence for the presence of recombination. The method is particularly powerful when the level of divergence between sequences is very low, as is the case with SARS-CoV-2 data, although it appears prone to false positives in the presence of severe mutation rate heterogeneity along the genome (Posada and Crandall 2001). We calculated an empirical estimate $\tilde{P}$ of mutation density along the genome from SARS-CoV-2 data, which does not suggest the presence of extreme heterogeneity, and then used this estimate to simulate the distribution of the number of recurrent mutations that are observed in a sample. The resulting probabilities and corresponding *P* values are shown in the third and fourth columns of table 1*a*–*d*.

### Validation on Simulated Data

#### False Positives due to Presence of Highly Homoplasic Sites

The accuracy of the presented method depends on an assumption that there are no highly homoplasic sites (arising either due to selection or repeated sequencing errors) that have not been masked. If this assumption were violated, the estimate $\tilde{P}$ would be missing “spikes” of high probability at the corresponding positions, biasing the simulated null distribution to underestimate the number of recurrent mutations, and potentially leading to false positive results.

**Fig. 1.**
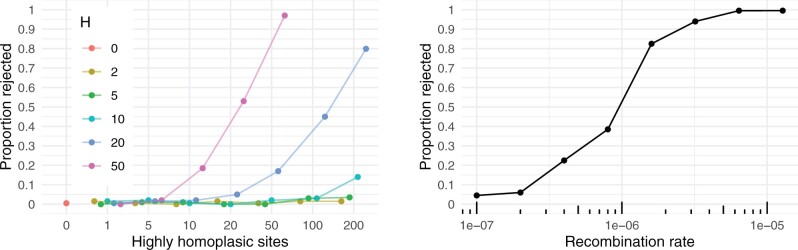
Left panel: *x*-axis shows number of added highly homoplasic sites, with the corresponding entries of $\tilde{P}$ multiplied by the factor *H* (colors); *y*-axis shows the proportion of simulated data sets (out of 200 for each combination of parameters) for which the null hypothesis was (incorrectly) rejected with *P*< 0.05. Right panel: *x*-axis shows recombination rate (per site per generation) used to simulate 200 data sets, *y*-axis shows proportion of data sets for which the null hypothesis was rejected with *P*< 0.05.

We investigated the validity of this assumption through simulation as described in supplementary section S4.3.2, Supplementary Material online, by inflating the mutation probability of a subset of 0–200 sites in the vector $\tilde{P}$ by a factor *H*, simulating data with the resulting mutation rate map in the absence of recombination with parameters that appear reasonable for SARS-CoV-2, and checking whether our method would (incorrectly) reject the null hypothesis. The results are presented in the left panel of figure 1.

False positives were seen in only 0.5% of cases when there are no highly homoplasic sites, demonstrating that our method conservatively overestimates the computed *P* values. The proportion of false positives only increases significantly when a large number of extremely homoplasic sites is present, showing that our method is reasonably robust to violations of this assumption. As we apply several stringent quality filters and implement a conservative strategy in masking sites known to be homoplasic, seeing a large number of extremely hypermutable sites appears improbable, so our method is unlikely to falsely indicate the presence of recombination.

#### Random Site-Level Variation in Mutation Rate

We also investigated the robustness of the method to random variation in the site-level mutation rates, which can arise for various biological reasons, such as the effects of selection on specific codons. First, as described in detail in supplementary section S4.4.1, Supplementary Material online, we simulated data sets under a model where sites have Gamma-distributed mutation rates (keeping the simulation parameters reasonable for SARS-CoV-2), then used our method to approximate the corresponding null distribution, and explored how the false positive rate changes as the variance of the Gamma distribution increases (while the mean stays fixed at $2\times 10^{-5}$ per site per generation). We found that the false positive rate remains low for reasonable values of the parameters (with the standard deviation of the site-level mutation rate per generation roughly equal to its mean of $2\times 10^{-5}$), and increases as the variance of the site-level mutation rate distribution grows.

This suggests that the method may not be suitable in cases where only a small fraction of sites have nonnegligible mutation rates. Although this does not appear to be the case for SARS-CoV-2, based on the observed number of segregating sites, we further checked robustness of the reported results using a phylogeny-based estimate of mutation rate heterogeneity. We used a SARS-CoV-2 phylogeny from Nextstrain (Hadfield et al. 2018; Sagulenko et al. 2018) to fit a Gamma distribution to site-level mutation rates, based on the observed site-level mutation count (as described in supplementary section S4.4.2, Supplementary Material online). We re-estimated the null distribution for each of the SARS-CoV-2 samples in table 1, using the Gamma approximation instead of our estimate of mutation rate heterogeneity $\tilde{P}$. In all cases, the calculated *P* values were below the significance threshold of 0.05, so we did not find that using this alternative phylogeny-based method changes our findings.

#### Detectable Recombination Rate

The power of our test in detecting the presence of recombination was investigated for a range of recombination rates *ρ*, by simulating data sets as described in supplementary section S4.3.3, Supplementary Material online, and recording how often the null hypothesis of no recombination could be rejected (with *P*< 0.05). The results are shown in the right panel of figure 1, demonstrating that this occurred in 4.5% of cases for $\rho =1\times 10^{-7}$ per site per generation, rising to 99.5% of cases for $\rho =1\times 10^{-5}$. The simulations were performed using parameters that appear reasonable for SARS-CoV-2; the results suggest that our method is sufficiently powerful for detecting recombination if the recombination rate is higher than around $\rho =1\times 10^{-6}$ per site per generation $\approx 4\times 10^{-5}$ per site per year.

### Identification of Recombinant Sequences

All sequences collected in England in December 2020–January 2021, labeled as belonging to clade GR in GISAID, were downloaded and processed as described in supplementary section S1.6, Supplementary Material online. The resulting sample comprises 40 sequences with 276 variable sites.

An illustration of the sample is provided in supplementary figure S6, Supplementary Material online. Choosing a solution with no recombinations, the sites of 14 recurrent mutations identified by KwARG are highlighted with red (resp. yellow) crosses, where the recurrent mutations fall on the terminal (resp. internal) branches of the ARG. The sequencing protocol used by the COVID-19 Genomics UK Consortium, the submitters of the data, generates short amplicons of under 400 bp in length, and none of the identified sites of recurrent mutations fall into the same amplicon region, making it less likely that the results are due to sample contamination or other sequencing artifacts. The probability of observing the required *T*_obs_ = 14 or more recurrent mutations is $p=1\times 10^{-6}$, which strongly indicates the presence of recombination.

**Fig. 2.**
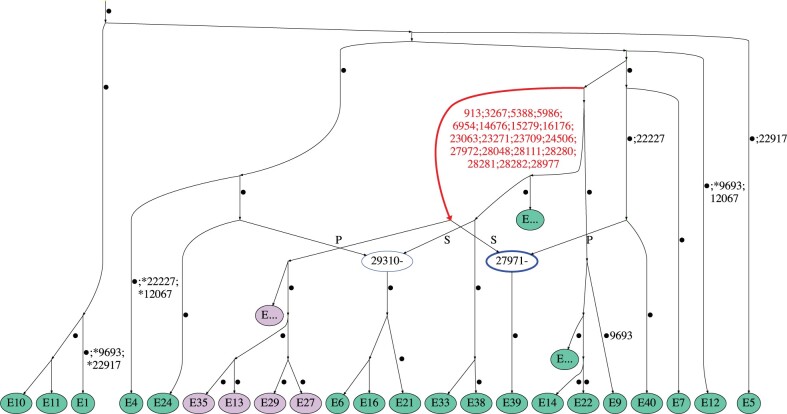
Example of an ARG for the England (January) data set. Recombination nodes are shown in blue, labeled with the recombination breakpoint, with the offspring sequence inheriting part of the genome to the left (right) of the breakpoint from the parent labeled “P” (“S”). Recurrent mutations are prefixed with an asterisk. Edge carrying the characteristic mutations of lineage B.1.1.7 is highlighted in red; nodes corresponding to sequences from lineage B.1.1.7 are colored purple. For ease of viewing, some parts of the ARG have been collapsed into nodes labeled “E….” Edges are labeled by positions of mutations (some mutated sites are not explicitly labeled and are denoted by a dot instead).

Considering the results in table 1*c*, three recurrent mutations can have the same effect as six of the identified recombination events (compare row (R,RM)=(10,0) with (R,RM)=(4,3)), suggesting that recurrent mutation offers a more parsimonious explanation for at least part of the patterns seen in the data. One of these recurrent mutations consistently occurs at site 22,227; the other two can be placed either at the same site 9,693, or at sites 9,693 and 12,067. The probability of observing five or fewer recurrent mutations is 0.97, which suggests that, with high probability, at least two recombination have occurred in the history of the sample. An example of an ARG with two recombination events is shown in figure 2.

It is striking that eight of the recurrent mutations seen in supplementary figure S6, Supplementary Material online, can be placed in the same sequence E39. Indeed, figure 2 shows that the corresponding incompatibilities in the data can be resolved by just one recombination event between sequence E40 and a sequence from lineage B.1.1.7; the corresponding recombination node is shown in bold. The sequence E39 has previously been identified as a possible recombinant by Jackson et al. (2021), demonstrating that our method can clearly highlight mosaic sequences in addition to quantifying the probability that recombination has occurred in the history of the data set.

### Detection of Intraclade Recombination

All sequences collected in South Africa in February 2021 were downloaded and processed as described in supplementary section S1.4, Supplementary Material online. The resulting sample comprises 38 sequences with 151 variable sites, all from the same lineage B.1.351.

Initial examination of the solutions identified by KwARG show that at least eight recurrent mutations are required to construct a valid ARG for this sample in the absence of recombination. However, it was noted that three of these recurrent mutations occur at the same site 28,254. This may imply that the site is highly mutable, which could be due to repeated sequencing errors, or as a consequence of selection. We note that this demonstrates the usefulness of our approach in identifying potentially highly homoplasic sites.

This position was masked from the sample before rerunning the analysis. The probability of observing the recalculated value of *T*_obs_ = 5 or more recurrent mutations is $P=7\times 10^{-4}$, strongly suggesting the presence of recombination. The probability of observing two or fewer recurrent mutations is 0.97, which indicates that with high probability, at least three recombination events have occurred in the history of the data set.

### Detection of Interclade Recombination

All sequences collected in South Africa in November 2020 were downloaded and processed as described in supplementary section S1.3, Supplementary Material online, to create a sample of 50 sequences with 207 variable sites, with 25 belonging to lineage B.1.351 (labeled SAN1-SAN25), and 25 to other lineages (labeled SAO1-SAO25).

An initial run of KwARG demonstrated that, notably, one recurrent mutation occurs at site 28,254, further suggesting that this site is excessively prone to recurrent mutation. This site was therefore masked before rerunning the analysis. An illustration of the sample is provided in supplementary figure S7, Supplementary Material online. The sites of nine recurrent mutations identified by KwARG are highlighted with red crosses (choosing a solution with no recombinations, and where the recurrent mutations fall on the terminal branches of the ARG). The probability of observing the required *T*_obs_ = 9 or more recurrent mutations is $P=7\times 10^{-6}$, strongly suggesting the presence of recombination.

**Fig. 3.**
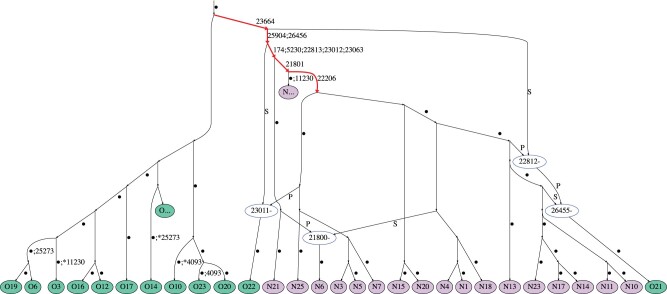
Example of an ARG for the South Africa (November) data set (the “SA” prefix of each sequence reference number is dropped for ease of viewing). Recombination nodes are shown in blue, labeled with the recombination breakpoint, with the offspring sequence inheriting part of the genome to the left (right) of the breakpoint from the parent labeled “P” (“S”). Recurrent mutations are prefixed with an asterisk. For ease of viewing, some parts of the ARG have been collapsed into nodes labeled “O…” and “N…” (containing sequences labeled SAO and SAN, respectively). Edges are labeled by positions of mutations (some mutated sites are not explicitly labeled and are denoted by a dot instead).

The probability of observing three or fewer recurrent mutations is 0.96, which indicates that, with high probability, at least four recombination events have occurred in the history of the data set. Indeed, table 1 shows that three recurrent mutations can remove the necessity of six recombination events, suggesting that recurrent mutation offers a more parsimonious explanation than recombination for the remaining incompatibilities in the data. Examination of the KwARG solutions shows that these recurrent mutations consistently occur at sites 4,093, 11,230, and 25,273. An ARG with recurrent mutations at these three sites is shown in figure 3; edges carrying the characteristic mutations of lineage B.1.351 are highlighted in red.

**Fig. 4.**
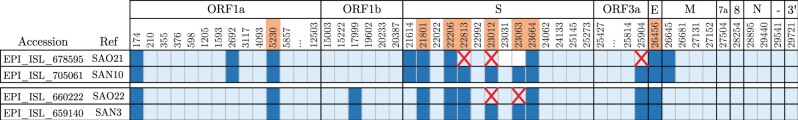
Comparison of sequences SAO21, SAO22 and the characteristic mutations for lineage B.1.351. Columns correspond to positions along the genome; uninformative sites (with all 0’s or 1’s) and those with singleton mutations (with exactly one 1) are not shown. Light blue: ancestral state, dark blue: mutated state, white: missing data. Red crosses highlight sites of recurrent mutations identified by KwARG. Sites bearing the characteristic (nonsynonymous) mutations of lineage B.1.351 (Tegally et al. 2021) are highlighted in orange.

The sequences SAO21 and SAO22 carry three and two of the identified nine recurrent mutations, respectively, when recombination is prohibited in reconstructing the genealogy. Both of these sequences carry some of the mutations characteristic of lineage B.1.351; this is demonstrated in figure 4, where the two sequences are compared with two other typical sequences from lineage B.1.351. Examination of the KwARG solutions shows that a recombination in Sequence SAO21 just after site 22,812 has the same effect as the recurrent mutations at sites 22,813 and 23,012, and a recombination in Sequence SAO22 just after site 23,011 has the same effect as the recurrent mutations at sites 23,012 and 23,063. This suggests that the patterns of incompatibilities observed in these two sequences are consistent with recombination; a possible sequence of recombination events generating these sequences can be seen in the ARG in figure 3.

### Identification of Sequencing Errors due to Cross-Contamination

All sequences labeled as GISAID clade GR, collected in England in November 2020, were aligned, masked, and processed as detailed in supplementary section S1.5, Supplementary Material online. The quality criteria detailed in supplementary section S1.2, Supplementary Material online, were *not* applied in this case. The resulting sample comprises 80 sequences with 363 variable sites, 40 of which belong to lineage B.1.1.7 (labeled EN1-EN40) and 40 to other lineages (labeled EO1-EO40).

**Fig. 5.**
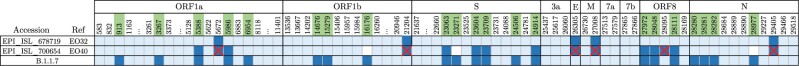
Comparison of sequences EO32, EO40 and the characteristic mutations of lineage B.1.1.7. Columns correspond to positions along the genome; uninformative sites (with all 0’s or 1’s) and those with singleton mutations (with exactly one 1) are not shown. Light blue: ancestral state, dark blue: mutated state, white: missing data. Red crosses highlight locations of the recurrent mutations identified by KwARG. Sites bearing the characteristic mutations of lineage B.1.1.7 (Rambaut et al. 2020) are highlighted in green.

The results showed that in the absence of recombination, at least 15 recurrent mutations were required to explain the incompatibilities observed in this sample. However, it was identified that six of these recurrent mutations could be placed in the same sequence EO40, as illustrated in figure 5. The sequence EO40 appeared to carry some of the mutations carried by sequence EO32, and some of the mutations characteristic of lineage B.1.1.7, strongly suggesting that this sequence was a recombinant.

Our findings prompted further investigation by the submitters of this sequence, which revealed the signal to be the result of significant contamination of the genetic sample causing multiple errors in the consensus sequence, rather than a result of intrahost recombination. The sequence has subsequently been removed from GISAID.

### Recombination Detection for MERS-CoV Data

MERS-CoV sequences collected in Saudi Arabia in January–March 2015 were downloaded from the NCBI virus database (Hatcher et al. 2017), and aligned, masked, and processed as described in supplementary section S2, Supplementary Material online. The resulting sample consists of 19 sequences with 197 variable sites.

The data set is illustrated in supplementary figure S8, Supplementary Material online. The locations of recurrent mutations identified by KwARG are shows as red and yellow crosses, corresponding to recurrent mutations occurring on the terminal and internal branches of the ARG, respectively. In the absence of recombination, at least *T*_obs_ = 16 recurrent mutations are required, which has probability $P<1\times 10^{-6}$, strongly suggesting the presence of recombination. The probability of observing three or fewer recurrent mutations is 0.99, suggesting that at least five recombinations have occurred in the history of the sample. An ARG with five recombination nodes, showing a possible history of the data set, is shown in supplementary figure S9, Supplementary Material online.

A group of four identical sequences (M16–M19, shown in purple in supplementary figure S9, Supplementary Material online) appear to carry a characteristic set of shared mutations that strongly differentiates them from the other sequences in the sample. Five of the identified recurrent mutations affect this group, occurring in a relatively short stretch of the genome, suggesting that these patterns are indicative of recombination with other sequences in the sample carrying these mutations.

Five of the other identified recurrent mutations can be placed in one sequence (M11), which appears to carry a mixture of mutations from the group identified above and other sequences in the sample, which is consistent with recombination. This sequence does not match any others in the data set, so it is possible that this is the result of sequencing errors or sample contamination. If this sequence is removed from the sample, at least *T*_obs_ = 9 recurrent mutations are still required to explain the observed incompatibilities, which has probability $P<1\times 10^{-6}$, still strongly suggesting that recombination is present. This agrees with previous reports of within-host recombination for MERS-CoV (Dudas and Rambaut 2016; Sabir et al. 2016; Zhang et al. 2016).

## Discussion

The method presented in this article offers a clear and principled framework for recombination detection, which can be interpreted as a hypothesis testing approach. We make very conservative assumptions throughout, demonstrating on both real and simulated data that the method achieves a very low rate of false positive results (if mutation rate heterogeneity is not extreme), while offering powerful detection of recombination at even relatively low values of recombination rate. We use nonparametric techniques at each stage, to avoid making assumptions on the process generating the data, and thus circumvent issues with model misspecification. Our method allows us to gain clear insights into the evolutionary events that may have generated the given sequences, offering easily interpretable results. The method detects sequences carrying patterns consistent with recombination, demonstrating its effectiveness as a tool for flagging sequences with distinctive patterns of incompatibilities for further detailed investigation.

Our results clearly indicate the presence of recombination in the history of the analyzed SARS-CoV-2 sequencing data, suggesting a recombination rate greater than around $4\times 10^{-5}$ per site per year. One of the main limitations of our method is that KwARG does not scale well to large data sets. However, although studies relying on clade assignment and statistics such as linkage disequilibrium have identified that recombination occurs at very low levels (VanInsberghe et al. 2021; Varabyou et al. 2021) or is unlikely to be occurring at a detectable level (De Maio et al. 2020; Nie et al. 2020; Richard et al. 2020; Tang et al. 2020; Wang et al. 2020; van Dorp, Richard, et al. 2020) even when analyzing vast quantities of sequencing data, our method is powerful enough to detect the presence of recombination using even relatively small samples. Several alternative methods are available for reconstructing genealogies explicitly in the presence of recombination, both with (Lyngsø et al. 2005) and without (Rasmussen et al. 2014; Kelleher et al. 2019; Speidel et al. 2019) making the parsimony assumption, but none is tailored to the particular problem of detecting recombination in the presence of recurrent mutation. Our testing framework could potentially be used in combination with these other methods for reconstructing ARGs, with appropriate modifications to control the false positive rate and ensure validity of the results.

Recombination can occur when the same host is coinfected by two different strains, which has been noted to occur in COVID-19 patients (Samoilov et al. 2021), and could become more likely with the emergence of more transmissible variants. We note that the potential mosaic sequences we identified in the South Africa sample from November are represented only once in the data. This could be due to a lack of onward transmission, as recombinants are likely to reach a detectable level at a relatively late stage in the infection cycle. It could also indicate that the sequences arose due to either contamination of the sample during processing, or the misassembly of two distinct (nonrecombinant) strains present in the same sample, as was identified to be the case for one sequence in the England sample from November. We believe one of the main uses of our method will be for flagging such sequences for further investigation.

We also note that although we sought to mask any sites known to be highly homoplasic, we cannot rule out that some of the identified recurrent mutations did arise multiple times as a consequence of selection or as a result of repeated sequencing errors. However, we have demonstrated that the solutions presented by KwARG can be examined for the presence of highly mutable sites, and have identified using both samples from South Africa that this appears to be the case for site 28,254 (located proximal to the stop codon of ORF8).

Our findings suggest that care should be taken when performing and interpreting the results of analysis based on the construction of phylogenetic trees for SARS-CoV-2 data. The presence of recombination, as well as other factors complicating the structure of the transmission network of the virus, strongly suggests that tree-based models are not appropriate for modeling SARS-CoV-2 genealogies, and inference of evolutionary rates based on such methods may suffer from errors due to model misspecification that are difficult to quantify.

Due to the high level of homogeneity between sequences, the effects of recombination will be either undetectable or indistinguishable from recurrent mutation in the majority of cases. However, as genetic diversity builds up over longer timescales, the effects of recombination may become more pronounced. Particularly in light of the recent emergence of new variants, the rapid evolution of the virus through recombination between strains with different pathogenic properties is a crucial risk factor to consider. This highlights the need for continuous monitoring of the sequenced genomes for new variants, to enable the early detection of novel recombinant genotypes, and for further work on the quantification of recombination rates and identification of recombination hotspots along the genome.

## Materials and Methods

### SARS-CoV-2 Data

Sequences were downloaded from GISAID and aligned as described in supplementary section S1.1, Supplementary Material online. Masking was applied to sites at the endpoint regions of the genomes, any multiallelic sites, regions with many missing nucleotides in multiple sequences, and sites identified by De Maio et al. (2020) as being highly homoplasic or prone to sequencing errors. Strict quality criteria were applied, as detailed in supplementary section S1.2, Supplementary Material online, to remove any sequences with a large number of ambiguous nucleotides, multiple non-ACTG characters, excessive gaps, and groups of clustered SNPs; additionally, sites identified by van Dorp, Acman, et al. (2020) as being prone to recurrent mutation were masked. These measures were aimed at reducing the possibility of including poor quality or contaminated sequences in the analyzed samples, and also masking sites that are known to be highly homoplasic (either due to recurring sequencing errors, or due to the effects of selection).

The timing of samples was selected to coincide with periods of high transmission numbers, as this increases the probability of coinfection of the same host with multiple strains, which is a requirement for recombination to occur. Collection dates were also restricted to reasonably narrow windows, as KwARG assumes that the sequences are sampled contemporaneously.

Four samples were analyzed; details of sample selection and processing are given in supplementary sections S1.3–S1.6, Supplementary Material online.

### Reconstruction of Genealogies

The first step in our approach is to use a parsimony-based method to reconstruct possible genealogical histories for the given data sets.

#### Incompatibilities in the Data

Suppose that each site of the genome can mutate between exactly two possible states (thus excluding the possibility of triallelic sites, which we have masked from the data). Then the allele at each site can be denoted 0 or 1. If the commonly used *infinite sites* assumption is applied, at most one mutation can affect each site of the genome. The *four gamete* test (Hudson and Kaplan 1985) can then detect the presence of recombination: if all four of the configurations 00, 01, 10, 11 are found in any two columns, then the data could not have been generated through replication and mutation alone, and at least one recombination event must have occurred between the two corresponding sites; the sites are then termed *incompatible*. If the infinite sites assumption is violated, the four gamete test no longer necessarily indicates the presence of recombination, as the incompatibilities could instead have been generated through recurrent mutation (McVean et al. 2002).

#### Parsimonious Reconstruction of Histories

A sample of genetic sequences may have many possible histories, with many different corresponding ARGs. The *parsimony* approach to reconstructing ARGs given a sample of genetic data focuses on minimizing the number of recombination and/or recurrent mutation events. This does not necessarily produce the most biologically plausible histories, but it does provide a lower bound on the number of events that must have occurred in the evolutionary pathway generating the sample. Thus, recombination can be detected in the history of a sample by considering whether the most plausible parsimonious solutions contain at least one recombination node.

#### KwARG

KwARG (Ignatieva et al. 2021) is a program implementing a parsimony-based heuristic algorithm for reconstructing plausible ARGs for a given data set. KwARG identifies “recombination only” solutions (all incompatibilities are resolved through recombination events) and “recurrent mutation only” solutions (all incompatibilities are resolved through additional mutation events), as well as interpolating between these two extremes and outputting solutions with a combination of both event types. KwARG allows for missing data and disregards insertions and deletions (we have deleted insertions from the alignment and treat deletions as missing data). KwARG seeks to minimize the number of posited recombination and recurrent mutation events in each solution, and the proportions of the two event types can be controled by specifying “cost” parameters. KwARG was run on the data samples as detailed in supplementary section S3, Supplementary Material online.

### Evaluation of Solutions

In order to evaluate which of the solutions identified by KwARG is more likely, we calculate an estimate $\tilde{P}$ which captures the mutation rate heterogeneity along the genome, and use a simulation-based approach to estimate the probability of observing a given number of recurrent mutations in the history of a given data set.

**Fig. 6.**
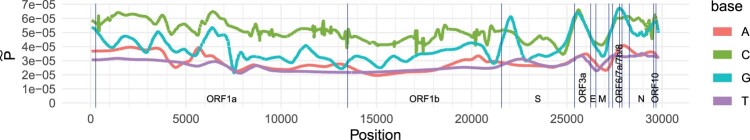
Estimate $\tilde{P}$ of the probability of a mutation falling on each site of the SARS-CoV-2 genome. Colors show the nucleotide type at each position. Blue vertical lines mark endpoints of the labeled ORFs and genes as per Wu et al. (2020).

The *i*th entry of the vector $\tilde{P}$, for i∈{1,…,29,903}, gives an estimated probability that when a mutation occurs, it affects the *i*th site of the genome. Details of our method for estimating $\tilde{P}$ are presented in supplementary section S4, Supplementary Material online. Briefly, this estimate is calculated by examining the locations of sites that have undergone at least one mutation (segregating sites) using GISAID data collected in February 2021. If the mutation rate were constant along the genome, we would expect segregating sites to be spread uniformly throughout the genome; uneven clustering of the mutations gives an indication of mutation rate heterogeneity. We use a nonparametric method (wavelet decomposition) to estimate $\tilde{P}$ from the observed positions of segregating sites, taking into account the dependence of the mutation rate on the base type of the nucleotide undergoing mutation, which is significant for SARS-CoV-2 (Koyama et al. 2020; Simmonds 2020). The resulting estimate is shown in figure 6.

The estimate of $\tilde{P}$ is then used to approximate the distribution of the number of recurrent mutations observed in a sample, using a simulation approach. We simulate the process of mutations falling along the genome until the simulated number of segregating sites matches that observed in the sample; the vector $\tilde{P}$ controls where on the genome each mutation falls. The number of recurrent mutations (instances where mutations fall on the same site multiple times) is recorded. This procedure is repeated for 1,000,000 iterations and a histogram of the results is constructed. The resulting probabilities and corresponding *P* values are shown in the third and fourth columns of table 1*a*–*d*.

## Supplementary Material

Supplementary data are available at *Molecular Biology and Evolution* online.

## Code Availability

Code used in processing the data (with step-by-step instructions for carrying out the analysis) is available at github.com/a-ignatieva/sars-cov-2-recombination.

## Supplementary Material

msac028_Supplementary_Data
