# Supplementary material for: Ongoing Recombination in SARS-CoV-2 Revealed through Genealogical Reconstruction
Source: Mol Biol Evol. 2022 Feb 2;39(2):msac028. doi: 10.1093/molbev/msac028 (PMC8841603; doi:10.1093/molbev/msac028)
Supplement: msac028_Supplementary_Data [file msac028_supplementary_data.zip › supplementary_information.pdf]

ONGOING RECOMBINATION IN SARS-CoV-2 REVEALED THROUGH GENEALOGICAL RECONSTRUCTION

ANASTASIA IGNATIEVA[1], JOTUN HEIN[2 4], AND PAUL A. JENKINS[1 3 4 *]

Supplemetary Information

[1] Department of Statistics, University of Warwick, Coventry CV4 7AL, UK
[2] Department of Statistics, University of Oxford, 24-29 St Giles', Oxford OX1 3LB, UK
[3] Department of Computer Science, University of Warwick, Coventry CV4 7AL, UK
[4] The Alan Turing Institute, British Library, London NW1 2DB, UK
[*] *E-mail*: p.jenkins@warwick.ac.uk

Supplementary Information

S1. Data: SARS-CoV-2

S1.1. **Alignment and masking.** SARS-CoV-2 sequences were downloaded from GISAID (Elbe & Buckland-Merrett, 2017), filtering for those labelled as complete (>29 000bp), collected from human hosts, and excluding any with more than 5% ambiguous nucleotides and incomplete collection dates. Although SARS-CoV-2 is an RNA virus, we refer to nucleotides by their DNA type for consistency with the sequencing data (i.e. the base type T corresponds to U on the actual SARS-CoV-2 genome).

Alignment to the reference sequence collected in Wuhan in December 2019 (Wu *et al.*, 2020) (GISAID accession: EPI_ISL_402125, GenBank: MN908947.3) was performed using MAFFT v7.475 (Katoh & Standley, 2013), with the options: `auto`, `keeplength`, `preservecase`, `addfragments`.

The following sites were masked from the data:

- the endpoint regions with a large number of missing nucleotides (1–55bp and 29 804–29 903bp);
- 322 further sites identified as problematic by De Maio *et al.* (2020) (prone to sequencing errors, known to be excessively homoplasic, or otherwise of questionable quality);
- any multi-allelic sites.

S1.2. **Quality criteria.** Any sequences failing the following quality criteria were removed:

- at most 500 missing nucleotides (excluding start and end of alignment);
- at most 1 non-ACTG character;
- at most 25 gaps;
- no SNP clusters (more than 6 SNPs in a window of 100 nucleotides, excluding known clusters of mutations).

Nextclade (Hadfield *et al.*, 2018, tool available at clades.nextstrain.org) was used to check sampled sequences against these criteria (and it was ensured that any sequences assigned a score of "bad" by the tool were removed). In addition, the 198 sites identified by van Dorp *et al.* (2020, Supplementary Table S5) as potentially highly homoplasic were masked.

S1.3. **South Africa (November).** All sequences collected in South Africa in November 2020 were downloaded and aligned as described in Section S1.1. Removing 48 sequences flagged by the submitter as containing long stretches of ambiguous nucleotides, and applying the quality criteria in Section S1.2, left a total of 278 sequences.

The aligned sequences were split into the datasets SA_N (the 177 sequences labelled as belonging to variant 501Y.V2 in GISAID) and SA_O (the other 101 sequences). A sample of 25 sequences from each of SA_O and SA_N was selected at random using SeqKit (Shen *et al.*, 2016).

Masking was carried out as described in Section S1.1; in addition, sites 22 266–22 745 were masked, as many of the sequences contained a large number of ambiguous nucleotides at these positions. No further multi-allelic sites were identified. Of the total 1 125 masked positions, 28 corresponded to segregating sites in the dataset.

The resulting sample comprises 50 sequences with 207 variable sites. The corresponding GISAID accession numbers and collection dates are given in Table S1.

S1.4. **South Africa (February).** All sequences collected in South Africa in February 2021 were downloaded, aligned, and masked as described in Section S1.1, also masking sites 22 266–22 745; no additional multi-allelic sites were identified. The quality filters detailed in Section S1.2 were applied. One sequence in the resulting sample was not from lineage B.1.351 and was removed. Of the total 1 125 masked positions, 17 corresponded to segregating sites.

The resulting sample consists of 38 sequences, all from lineage B.1.351, with 151 variable sites. The corresponding GISAID accession numbers and collection dates are given in Table S2.

S1.5. **England (November).** All sequences labelled as clade GR, collected in England in November 2020, were downloaded and aligned as per Section S1.1. Exact duplicates of sequences in the dataset were removed, to avoid including identical sequences in the sample. The sequences were then split into datasets E_N (934 sequences labelled as belonging to lineage B.1.1.7) and E_O (the other 2 650 sequences).

A sample of 40 sequences from each of E_O and E_N was then selected at random using SeqKit. Sites were masked as detailed in Section S1.1. Three multi-allelic sites were identified and masked, at positions 12 067, 21 724, and 22 992. Of the total 477 masked positions, 10 corresponded to segregating sites in the dataset. The quality control criteria in Section S1.2 were *not* applied to this sample.

The resulting sample comprises 80 sequences with 363 variable sites. The corresponding GISAID accession numbers and collection dates are given in Table S3.

S1.6. **England (January).** All sequences labelled as clade GR, collected in England in December 2020 – January 2021, were downloaded and aligned as per Section S1.1. Sites were masked as detailed in Section S1.1, and the quality filters detailed in Section S1.2 were applied. A sample of 38 sequences was selected at random using SeqKit, from among sequences uploaded by the COG UK Consortium; additionally, the sequence EPI_ISL_994038 (E39) identified as a potential recombinant by Jackson *et al.* (2021), and its potential parent sequence EPI_ISL_820233 (E40), were included. Five multi-allelic sites were identified and masked, at positions 21 255, 23 604, 24 914, 28 310, and 29 227. Of the total 660 masked positions, 35 corresponded to segregating sites in the dataset.

The resulting sample comprises 40 sequences with 276 variable sites. The corresponding GISAID accession numbers and collection dates are given in Table S4.

S2. Data: MERS-CoV

MERS-CoV sequences were downloaded from the NCBI Virus database (Hatcher *et al.*, 2017), filtering for those labelled as complete, human host, collected in Saudi Arabia in January–March 2015. Alignment to the reference sequence (HCoV-EMC/2012, accession number NC_019843.3) was performed using MAFFT, with the same options as in Section S1.1. Masking of the first and last 150 sites of the alignment was performed. Of the 300 masked sites, two were segregating in the dataset; no multi-allelic sites were identified. The resulting sample consists of 19 sequences with 197 variable sites. The corresponding accession numbers are given in Table S5.

S3. KwARG

KwARG seeks to minimise the number of posited recombination and recurrent mutation events in each solution, and the proportions of the two event types can be controlled by specifying input 'cost' parameters C_{SE}, C_{RM}, C_R, and C_{RR}, corresponding to penalties assigned to recurrent mutations on the terminal branches of the ARG, those on internal branches, recombination events, and two consecutive recombination events (which can mimic the effects of gene conversion), respectively. For instance, setting $(C_{SE}, C_{RM}, C_R, C_{RR}) = (0.5, 0.51, 1.0, 2.0)$ is likely to produce solutions with more recurrent mutations than recombinations, as the cost of recurrent mutations is lower, favouring placing recurrent mutations on the terminal branches of the ARG where possible. Recurrent mutations on the terminal branches of the ARG affect only one sequence in the input dataset, so can be examined separately for indications that they arose due to errors in the sequencing process. Note that these parameters only control the exploration of the

space of plausible ARGs (and not the evaluation of the likelihood of each solution, which is implemented as a separate procedure).

KwARG implements a method of randomly exploring the space of possible ARGs, so it should be run multiple times for each configuration of input parameters, and the best identified solutions (with the minimal number of posited recombinations and/or recurrent mutations) then selected for analysis. An input parameter T (the 'annealing temperature') controls the extent of this random exploration.

S3.1. **Parameter settings.** For each dataset, KwARG was run $Q = 500$ times for each combination of the following values of the annealing parameter T and event costs $(C_{SE}, C_{RM}, C_R, C_{RR})$:

$$\begin{aligned} T &\in \{30, 50\} \qquad \text{(S1)} \\ (C_{SE}, C_{RM}, C_R, C_{RR}) &\in \{(\infty, \infty, 1, 2), (1.9, 1.91, 1, 2), (1.8, 1.81, 1, 2), (1.7, 1.71, 1, 2), \ldots \\ & (0.1, 0.11, 1, 2), (0.01, 0.02, 1, 2), (1.0, 1.1, \infty, \infty)\}. \end{aligned}$$

For MERS-CoV, the root was left unspecified. For SARS-CoV-2, the reference sequence used for alignment was set as the root. This reference sequence is a genome collected in Wuhan in December 2019 (Wu *et al.*, 2020), giving the most likely rooting based on the available epidemiological evidence; our results do not change significantly if the root is left unspecified.

S4. Null distribution for SARS-CoV-2

S4.1. **Distribution of the number of recurrent mutations.** Let M be the length of the genome, and let m be the number of observed variable sites in the sample. We are interested in estimating the distribution of the number of recurrent mutations that have occurred; that is, the excess number of mutation events beyond the minimum m needed to explain the variability in the sample.

Regardless of any modelling assumptions on the evolution of a given sample or the genealogical relationships between the sequences, it is clear that at least m mutation or sequencing error events must have occurred in the history of the sample (here, a 'sequencing error' refers to the variant at a site being incorrectly called during the sequencing process). Suppose that each time such an event occurs (disregarding which particular sequence is affected), a position on the genome is selected at random with replacement, according to a probability vector P of length M. This corresponds to assuming that (i) such events occur independently from each other, (ii) all sequences have the same probabilities P of a mutation or sequencing error event occurring at each particular site. Moreover we assume that (iii) if a site undergoes at least one mutation in the history of the sample, the site is segregating in the data; and (iv) any sequencing errors fall on each site with probability proportional to P. The validity of these assumptions is discussed below in Section S4.3.

The number of recurrent mutations in a sample with m variable sites can then be simulated using Algorithm 1. This is a 'balls-into-bins' type simulation, in which balls are placed one-by-one into M bins, each time selecting a bin at random with probability proportional to P, until m bins contain at least one ball; the output is the total number of balls thrown minus m. Executing Algorithm 1 multiple times and calculating a histogram of the results gives an approximation to the distribution of the number of recurrent mutations given the number m of observed segregating sites.

S4.2. **Mutation rate heterogeneity along the genome.** Parts of the genome with a relatively higher mutation rate are more likely to undergo recurrent mutation, so it is important to incorporate the effects of mutation rate heterogeneity. We use an empirical estimate of mutation density to approximate the variation in mutation rate along the genome.

Algorithm 1: Simulating the number of recurrent mutations conditional on observing m variable sites

```
Input: M, m, P
Output: Number of recurrent mutations m̃
Initialise m̃ = 0, S = {∅};
while |S| < m do
    Draw s from {0, ..., M} with probabilities proportional to P;
    if s ∉ S then
        S ← S ∪ s;
    end
    m̃ ← m̃ + 1 ;
end
m̃ ← m̃ − m ;
return m̃;
```

S4.2.1. *Data.* All 17 908 sequences in GISAID collected around the world between 1 and 3 February 2021 were downloaded, filtering for sequences labelled as complete (>29 000bp), high coverage, and excluding any with more than 5% ambiguous nucleotides. Alignment was performed as described in Section S1.1. SNP-sites (Page *et al.*, 2016) was used to extract the positions of the 13 747 identified SNPs; a vector $\overline{P}$ of length 29 903 was then formed, with a 1 entry at position i if there had been at least one mutation at position i of the genome, and 0 otherwise. If the mutation rate is constant along the genome, we would expect the 1's to be spread uniformly throughout $\overline{P}$; uneven clustering of the mutations gives an indication of mutation rate heterogeneity. We note that an alternative approach would be to fit a tree to the sequencing data (using maximum likelihood, for instance), count the minimum number of mutations required at each site of the genome, and use this to estimate P. However, this was found to result in very noisy estimates, and provide worse quantification of mutation rate heterogeneity (which we confirmed through simulation studies).

S4.2.2. *Smoothing.* The mutation density along the genome was then estimated nonparametrically from $\overline{P}$ by smoothing using wavelet decomposition, as implemented in the R package `wavethresh` (Nason *et al.*, 2010). This method was chosen as it does not require selecting a particular model, and it captures both fine-scale and broad variation in mutation density, allowing for the calculation of a smoothed estimate of $\overline{P}$ incorporating both local and large-scale rate heterogeneity.

Briefly, wavelet decomposition can be used to obtain an estimate of a signal from a set of discrete observations, by analysing variation in the data at increasingly coarser scales (Nason, 2008). Given $M = 2^n$ observations of sites, corresponding to the entries of $\overline{P}$ (padding the vector $\overline{P}$ to the nearest power of 2 by reflecting the data at the endpoints), n iterations are performed, and at the i-th iteration (1) coefficients are computed using (non-overlapping) subsets of 2^i neighbouring observations, and (2) these coefficients are used to refine a smoothed estimate of the data. The computation of coefficients and the smoothed approximations is governed by the choice of wavelet shape; we use Daubechies' least-asymmetric wavelets (Daubechies, 1988) with six vanishing moments (other choices of wavelet basis produced similar results). Wavelet *shrinkage* can be used to obtain a smoothed estimate of the observations and remove noise: coefficient selection is performed by only keeping coefficients with values above a certain threshold and setting the others to zero. There are myriad ways of calculating such a threshold (Nason, 2008); we apply the empirical Bayes method of Johnstone & Silverman (2005b) implemented in the R package `EbayesThresh` (Johnstone & Silverman, 2005a).

As the mutation rate is dependent on the base type of the nucleotide undergoing mutation (Simmonds, 2020; Koyama *et al.*, 2020), $\overline{P}$ was split into four parts by the corresponding base type in the reference sequence, and the wavelet decomposition and thresholding performed separately for each part before joining them back together. The resulting smoothed estimate $\widetilde{P}$ is shown in Figure 6. The total estimated mutation probability for each base type closely matches the actual proportion of mutations that fall on sites of each base type in the data, as desired. The smoothing method has clearly identified both localised and long-range variation in mutation density along the genome.

To check consistency of the results across time periods, data from September–November 2020 was also used to produce smoothed estimates of $\overline{P}$ (consisting of 41 376 sequences with 14 263 variable sites). The resulting estimate was found to agree closely to that obtained using the February data, so the latter was used in further analysis.

S4.3. **Validity of assumptions.** We now return to consider the validity of the assumptions stated in Section S4.1. Assumption (i) appears reasonable for the data at hand. Assumption (iii) can be violated if a mutation arising on a branch of the genealogy subsequently reverses through recurrent mutation: either on the same branch before it splits, or independently on every child branch subtending the mutation. We note that the probability of such events depends on the distribution of branch lengths in the genealogy; simulations using the standard coalescent model show that the probability of such events is small. Moreover, such events can never create incompatibilities in the data, so we can ignore their possibility for our purposes, as the solutions identified by KwARG will never include such recurrent mutation events.

Regarding assumption (ii), as the mutation rates depend on the base type, we cannot claim that all sequences have exactly the same probabilities P of mutating at each particular site, as this will depend on the nucleotides carried by the sequence. However, we estimate the effect of this violation to be negligible, given the relatively low overall rate of mutation for SARS-CoV-2.

To make our approximation even more conservative, we increase m by adding back the number of masked segregating sites (which are as stated in Sections S1.3 to S1.6), and further multiply the number of sites by a penalty factor of $F = 1.1$, which is justified in Section S4.3.1 below. Thus, we address assumption (iv) by noting that we have masked sites that are excessively prone to sequencing errors in the data, so correspondingly we decrease M by the number of masked sites and delete the corresponding entries from $\widetilde{P}$. It is then reasonable to assume that sequencing errors occurring at the non-masked sites fall at each site with the same probabilities as mutations. The effects of this assumption being violated are explored further in Section S4.3.2.

S4.3.1. *Choice of penalty factor.* As noted above, the number of segregating sites in the sample is multiplied by a penalty factor F before performing the simulations. This results in a larger number of recurrent mutations being simulated, skewing the distribution to the right and thus ensuring that the p-values calculated from the simulated distribution are reasonably conservative. This is necessary because, as with any regression method that aims to (partially) de-noise the data, there is a risk that the fitted curve underestimates the true mutation rate heterogeneity, which would result in the expected number of recurrent mutations being underestimated, leading to false positives.

The choice of $F = 1.1$ was validated through simulation studies. First, we simulate a "true" mutation rate map P_{true} across 29,903 sites, as a realisation of an autoregressive process. Then, we simulate 20 000 mutations falling on the genome (allowing sites to mutate multiple times), and re-create the vector $\overline{P}$ by marking which sites had (or had not) undergone at least one mutation. The method described in Section S4.2 is then applied to fit an estimated mutation density P_{fit}. Finally, 10 000 simulations of Algorithm 1 are used to get an estimate of the null distribution: first, using P_{true} with $m \in \{100, 300, 500\}$

sample segregating sites, then using P_{sim} with $m \cdot F$ sample segregating sites, for $F \in \{1.0, 1.1, 1.2, 1.3, 1.4, 1.5\}$.

This procedure was repeated 500 times for each combination of m and F. The results are presented in Figure S1. This demonstrates that without the penalty term, the fitted mutation density may indeed fail to capture all of the mutation rate heterogeneity that is present; for instance, when considering a sample with 300 segregating sites, in 46% of cases the 95th percentile of the simulated distribution will be lower than that of the true distribution. The results demonstrate that a value of $F = 1.1$ appears sufficient to negate this effect, without excessively increasing the false negative rate.

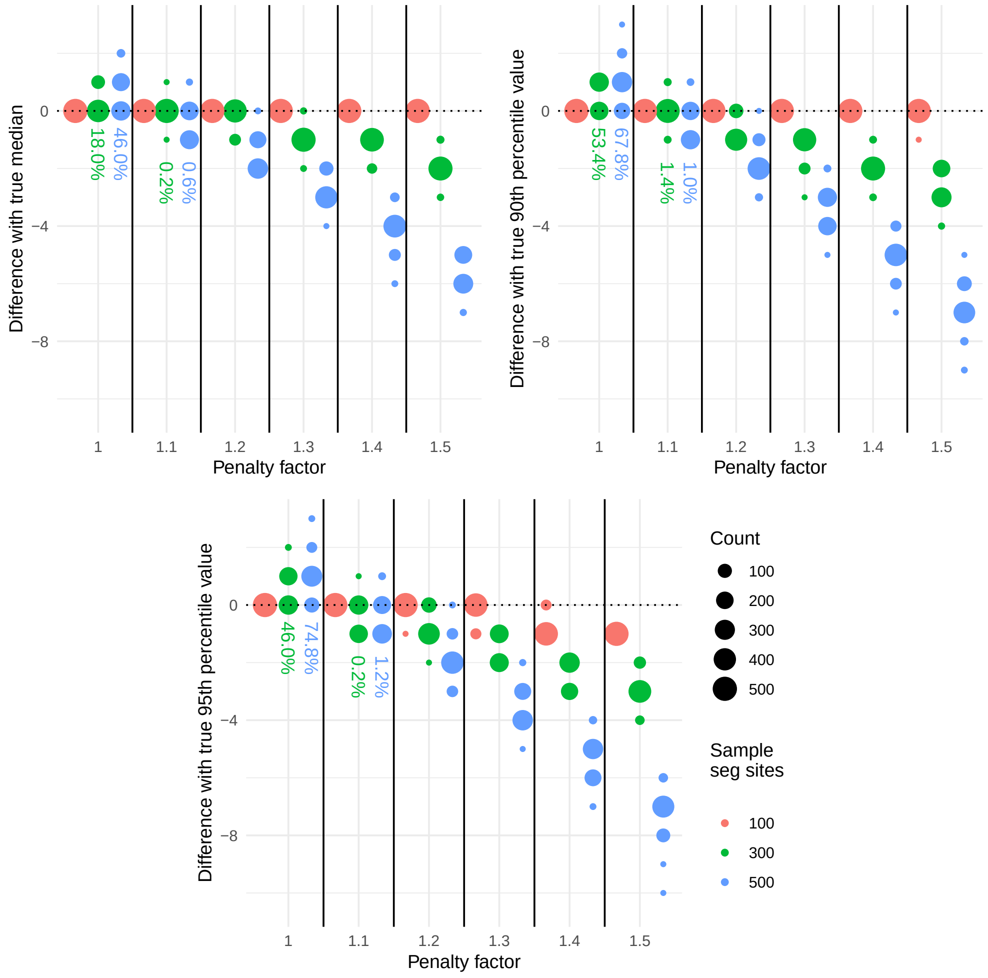

Figure S1. Comparison of simulated null distributions using P_{true} and P_{fit}. Points show the difference between the true and simulated median (left panel), 90th percentile (middle), 95th percentile (right), with size proportional to the number of observations, split by penalty factor F (x-axis) and the number of sample segregating sites m (colours). Ideally, the points should be concentrated around 0; values above (below) 0 may result in false positives (false negatives) when using the estimated null distribution. Percentages show the proportion of cases lying above 0.

S4.3.2. *Presence of highly homoplasic sites.* Violations of assumption (iv) can occur if some (non-masked) sites along the genome are highly homoplasic, which can occur due to the effects of selection, or as an artifact of the sequencing process. Our method will not

pick up the spikes in the corresponding positions of $\widetilde{P}$, potentially introducing bias to the simulated null distribution that could lead to false positives.

We investigate the extent to which a violation of assumption (iv) affects the resulting inference through simulation studies. For each $i \in \{0, 1, 5, 10, 20, 50, 100, 200\}$, i sites of the genome are chosen, and the corresponding probabilities in $\widetilde{P}$ are multiplied by a factor $H \in \{2, 5, 10, 20, 50\}$ to give the vectors $\widetilde{P}_{i,H}$. This recreates the effect of having i sites which are highly homoplasic (with the extent of this controlled by H); an example of $\widetilde{P}_{50,2}$ is shown in Figure S2.

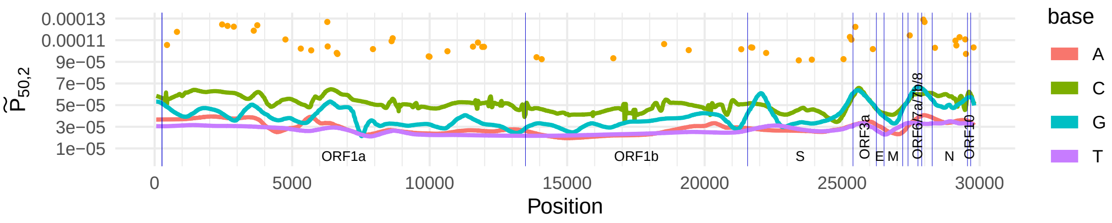

Figure S2. Mutation density estimate $\widetilde{P}$ adjusted by selecting $i = 50$ sites and multiplying the corresponding entry of $\widetilde{P}$ by $H = 2$ (resulting values shown in orange), to recreate the presence of 50 highly homoplasic sites. Colours show the nucleotide type at each site; sites with inflated mutation rates shown in orange. Blue vertical lines mark endpoints of the labelled ORFs and genes as per Wu *et al.* (2020).

For each combination of i and H, 200 datasets of 80 sequences were simulated using msprime (Kelleher *et al.*, 2016), with parameters that appear reasonable for SARS-CoV-2:

- $N_e = 1 \cdot 10^6$, exponential growth rate of 1.5 (no appropriate published estimates of these parameters could be identified, but this choice was found to give reasonable values of MRCA time and number of segregating sites for the simulated datasets);
- binary mutation model (finite sites);
- mutation rate per site per generation given by the entries of $\widetilde{P}_{i,H} \times 2 \cdot 10^{-5} \times 29\,903$. This was calculated based on:
  - a mean mutation rate of $8 \cdot 10^{-4}$ per site per year (as used by Nextstrain (Hadfield *et al.*, 2018), accessed through nextstrain.org/ncov/global);
  - a generation time of 7.5 days (Li *et al.*, 2020);
  - giving a mean mutation rate of $2 \cdot 10^{-5}$ per site per generation.

We note that some considerations that may affect viral genealogies were not incorporated into the model (such as multiple mergers and effects of spatial structure), both for simplicity and due to the difficulty in identifying realistic assumptions and parameter values.

For each dataset, KwARG was run 200 times (parameters: $T = 30$, $Q = 100$, $(C_{SE}, C_{RM}, C_R, C_{RR}) \in \{(1, 1.1, \infty, \infty), (0.01, 0.02, 1.00, 2.00)\}$) to calculate the minimal number of recurrent mutations needed to explain the dataset in the absence of recombination. A p-value was then calculated, using the null distribution simulated using the un-adjusted vector $\widetilde{P}$ and 10 000 iterations of Algorithm 1, with m set to the number of segregating sites in the dataset multiplied by the penalty factor $F = 1.1$.

The proportion of times the null hypothesis is (incorrectly) rejected, with $p < 0.05$, is shown in the left panel of Figure 1.

S4.3.3. *Detection rate vs recombination rate.* We investigate how the proportion of cases in which the null hypothesis is rejected (with $p < 0.05$) varies with recombination rate. For several values of the recombination rate $1 \cdot 10^{-7} \leq \rho \leq 1 \cdot 10^{-5}$ (per site per generation), 200 datasets were simulated using msprime with the parameters given in Section S4.3.2

(using the un-adjusted vector $\widetilde{P}$), and the same method used to calculate a p-value for each dataset. The results are presented in the right panel of Figure 1.

S4.4. **Further analysis of robustness.** The wavelet-based method described above produces a smooth estimate of the mutation rate heterogeneity along the genome, and we have demonstrated that the presence of additional highly homoplasic sites does not appear to be problematic. We now test the robustness of the method against site-level random variation in the mutation rate along the genome, which can arise due to multiple biological factors (such as genomic context effects, or selection acting on individual codons).

First, we simulate data under a model where the site-level mutation rates are Gamma-distributed, for varying values of the Gamma parameters, and estimate the false positive rate versus increasing variance of this distribution. Then, we use a phylogeny-based method to fit a Gamma distribution to observed SARS-CoV-2 site-level mutation rates, and rerun our analysis for the samples used to check if the null hypothesis of no recombination can be rejected using this alternative method.

S4.4.1. *Site-level random variation in mutation rate.* First, a mutation rate was drawn for each site of the genome, to create a vector $S = (s_1, s_2, \dots, s_{29\,903})$, with $s_i \sim^{iid} \Gamma(\alpha, \beta)$ (where α is the shape and β the scale parameter). Then, 20 000 mutations were simulated on the genome, and a vector $\bar{P}_S$ was constructed, with 1 entries at positions that had undergone at least one mutation, and 0 otherwise. The wavelet-based method described in Section S4.2.2 was used to fit a smoothed estimate $\widetilde{P}_S$ from $\bar{P}_S$ (without splitting up the data by nucleotide type). Then, msprime was used to simulate a sample (with settings and parameters as described in Section S4.3.2), with mutation rates S and a binary mutation model with direction switching from 0 to 1. This was repeated 200 times for each value of the parameters

$$(\alpha, \beta) \in \{(0.1, 2 \cdot 10^{-4}), (0.5, 4 \cdot 10^{-5}), (1.0, 2 \cdot 10^{-5}), (5.0, 4 \cdot 10^{-6}), (10.0, 2 \cdot 10^{-6}), (100.0, 2 \cdot 10^{-7})\}.$$

These parameter values were selected so that the mean mutation rate stayed fixed at $2 \cdot 10^{-5}$ per site per generation, while the variance ranged from $4 \cdot 10^{-9}$ to $4 \cdot 10^{-12}$. Plots of the distributions with this range of parameters are shown in Figure S4.

For each simulated sample, KwARG was run 200 times (parameters: $T = 30, Q = 100, (C_{SE}, C_{RM}, C_R, C_{RR}) \in \{(1.0, 1.1, \infty, \infty), (0.01, 0.02, 1.00, 2.00)\}$) to calculate the minimal number of recurrent mutations required explain the dataset in the absence of recombination. The null distribution was then estimated using 10 000 iterations of Algorithm 1, with m set to the number of segregating sites in the sample multiplied by the penalty factor $F = 1.1$, using the wavelet estimate $\widetilde{P}_S$. The proportion of times the null hypothesis was (incorrectly) rejected with $p < 0.05$ is shown against α in Figure S3.

The results show that, as can be expected, the false positive rate increases as the variance of the site-level mutation rate distribution increases: the wavelet-based estimate does not appropriately capture the truth when the genome consists of only a small number of highly mutable sites, and it is also possible that KwARG does not identify a tight enough lower bound in the given number of iterations when there are a lot of incompatibilities in the generated datasets. Reasonable accuracy is seen for moderate values of the parameters, when $\alpha \geq 1$ (corresponding to the mean and standard deviation of the mutation rate distribution being equal to $2 \cdot 10^{-5}$).

This suggests that the method will not perform well when α is very small and only a small fraction of the genome has a non-negligible mutation rate (which can be detected in practice by the presence of very few segregating sites). In this case, a more suitable alternative approach would be to simulate the null distribution using a fitted Gamma distribution for site-level mutation rates, instead of the wavelet-based estimator $\widetilde{P}$. To

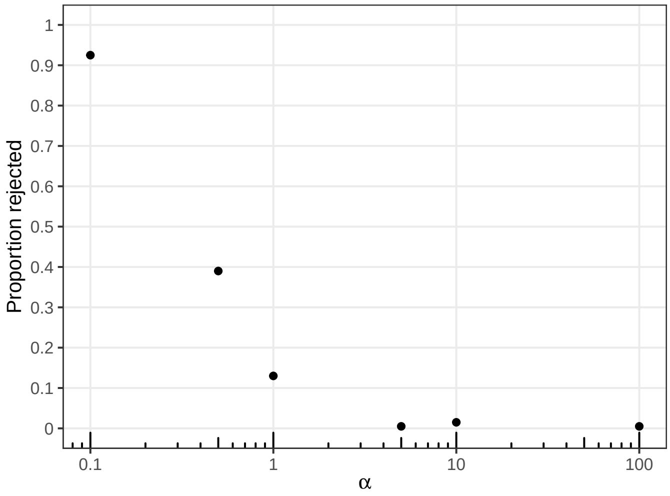

Figure S3. Proportion of simulations for which the null hypothesis was (incorrectly) rejected with $p < 0.05$, against the Gamma distribution shape parameter α (note that the scale parameter β is chosen so that the mean is fixed at $2 \cdot 10^{-5}$ for each choice of α)

further test robustness, we next check whether our results for the analysed SARS-CoV-2 samples remain significant using this alternative approach.

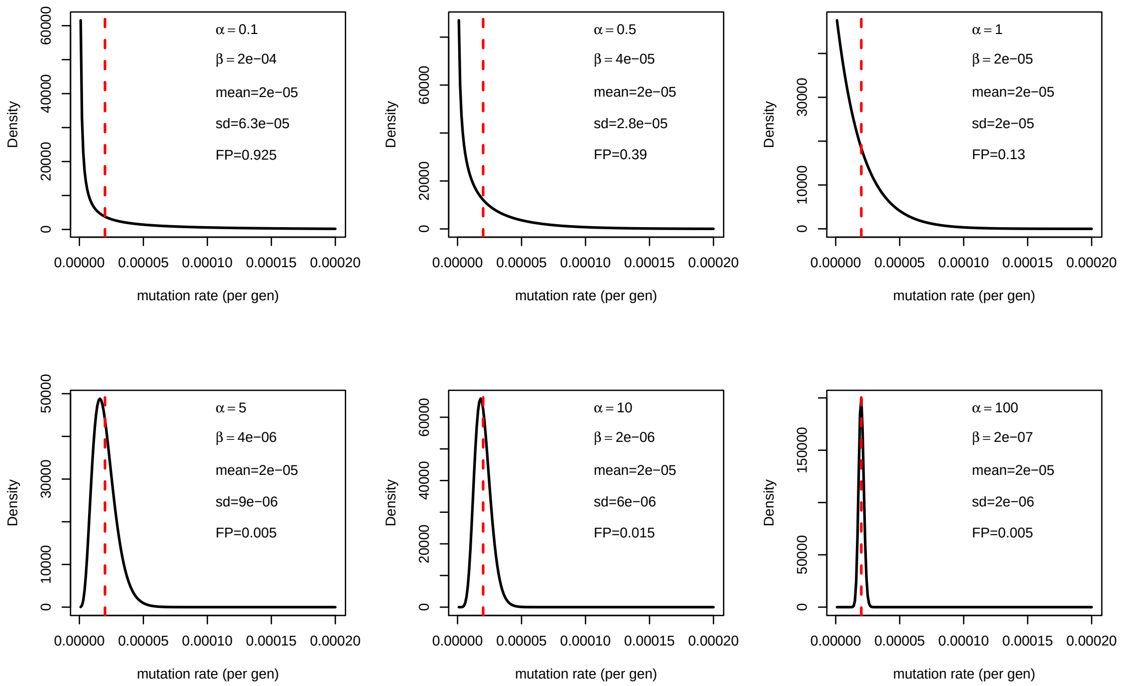

Figure S4. Simulated mutation rate distributions (α and β: shape and scale parameters of the Gamma distribution, respectively, chosen so that the mean is $\alpha \cdot \beta = 2 \cdot 10^{-5}$; FP = false positive rate; red dashed line shows the mean site-level mutation rate).

S4.4.2. *Comparison to phylogeny-based estimate of site-level mutation rates.* We now test whether an alternative approach of fitting a Gamma model to SARS-CoV-2 site-level mutation rates and using this to simulate the null distribution (instead of the wavelet-based estimator) changes the significance of the reported results for the analysed datasets.

We used the SARS-CoV-2 phylogeny provided by Nextstrain (Hadfield *et al.*, 2018; Sagulenko *et al.*, 2018), using data up to 21 November 2021, to get an estimate of the number of mutations that have occurred at each site of the genome. We fitted a Gamma-Poisson model to these mutation counts (including sites with zero mutations, and masking highly mutable and problematic sites as described in Sections S1.1 and S1.2), using the `fitdistrplus` R package (Delignette-Muller, Dutang, *et al.*, 2015), obtaining an MLE of $\widehat{\alpha} = 0.247$ and $\widehat{\beta} = 1.870$. The null distribution was then simulated for each of the SARS-CoV-2 samples in Table 1, using 10 000 iterations of Algorithm 1: the mutation rates were drawn i.i.d. from a $\Gamma(0.247, 1.870)$ distribution newly at each iteration, and each simulation was run until m segregating sites were reached (with m for each sample given in Section S6).

The calculated p-values were below 0.05 for all three datasets (South Africa (November): 0.016, South Africa (February): 0.049, England (January): 0.009), allowing for the hypothesis of no recombination to also be rejected in each case using this method. We note that the accuracy of this method will depend on the size and quality of the chosen phylogeny; the Nextstrain phylogeny was chosen as the analysis pipeline implements a number of quality control procedures, and filters the available SARS-CoV-2 data to choose a globally representative subsample of moderate size. We also did not attempt to recreate the effect of masking hypermutable sites, which would decrease the calculated p-values.

S5. Null distribution for MERS-CoV

The same methodology as described in Section S4 was used to simulate the null distribution for MERS-CoV.

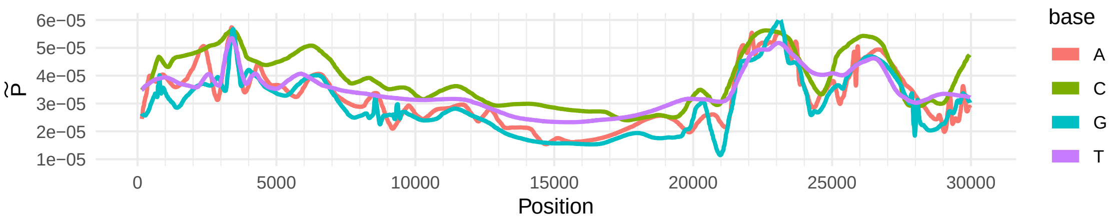

Figure S5. Estimate $\widetilde{P}$ of the probability of a mutation falling on each site of the MERS-CoV genome. Colours show nucleotide type at each position.

Sequences were downloaded from the NCBI Virus database, filtering for those of length at least 20 000 bp, from human and camel hosts, across all time periods. Alignment to the reference sequence was performed as described in Section S2. The alignment comprised 700 sequences with 14 238 variable sites. The vector $\overline{P}$ was constructed, and wavelet decomposition was used to fit the estimate $\widetilde{P}$ in the same manner as described in Section S4.2; the result is shown in Figure S5.

With the resulting estimate $\widetilde{P}$ and the parameters given in Section S6, 1 000 000 iterations of Algorithm 1 were used to simulate the null distribution. The resulting probabilities and p-values are shown in the third and fourth columns of Table 1.

S6. Null distribution simulation parameters

	SA (Nov)	SA (Feb)	England (Nov)	England (Jan)	MERS-CoV
No. of segregating sites	206	150	363	276	197
Plus masked sites	29	18	10	35	2
Times penalty factor F	1.1	1.1	1.1	1.1	1.1
m	259	185	410	342	219
Length of genome	29 903	29 903	29 903	29 903	30 119
Less number of masked sites	1126	1126	477	660	300
M (= length of $\widetilde{P}$)	28 777	28 777	29 426	29 243	29 819

References

1. Daubechies, I. Orthonormal bases of compactly supported wavelets. *Communications on Pure and Applied Mathematics* **41,** 909–996 (1988).
2. De Maio, N. *et al. Issues with SARS-CoV-2 sequencing data* `https://virological.org/t/issues-with-sars-cov-2-sequencing-data/473`. 2020.
3. Delignette-Muller, M. L., Dutang, C., *et al.* fitdistrplus: An R package for fitting distributions. *Journal of Statistical Software* **64,** 1–34 (2015).
4. Elbe, S. & Buckland-Merrett, G. Data, disease and diplomacy: GISAID's innovative contribution to global health. *Global Challenges* **1,** 33–46 (2017).
5. Hadfield, J. *et al.* Nextstrain: Real-time tracking of pathogen evolution. *Bioinformatics* **34,** 4121–4123 (2018).
6. Hatcher, E. L. *et al.* Virus Variation Resource–improved response to emergent viral outbreaks. *Nucleic Acids Research* **45,** D482–D490 (2017).
7. Jackson, B. *et al.* Generation and transmission of interlineage recombinants in the SARS-CoV-2 pandemic. *Cell.* doi:`10.1016/j.cell.2021.08.014` (2021).
8. Johnstone, I. M. & Silverman, B. W. EbayesThresh: R and S-Plus programs for empirical Bayes thresholding. *Journal of Statistical Software* **12,** 1–38 (2005).
9. Johnstone, I. M. & Silverman, B. W. Empirical Bayes selection of wavelet thresholds. *Annals of Statistics,* 1700–1752 (2005).
10. Katoh, K. & Standley, D. M. MAFFT multiple sequence alignment software version 7: Improvements in performance and usability. *Molecular Biology and Evolution* **30,** 772–780 (2013).
11. Kelleher, J., Etheridge, A. M. & McVean, G. Efficient coalescent simulation and genealogical analysis for large sample sizes. *PLoS Computational Biology* **12,** e1004842 (2016).
12. Koyama, T., Platt, D. & Parida, L. Variant analysis of SARS-CoV-2 genomes. *Bulletin of the World Health Organization* **98,** 495 (2020).
13. Li, Q. *et al.* Early transmission dynamics in Wuhan, China, of novel coronavirus–infected pneumonia. *New England Journal of Medicine* (2020).
14. Nason, G. *Wavelet methods in statistics with R* (Springer Science & Business Media, 2008).
15. Nason, G. *et al. Wavethresh: Wavelets statistics and transforms, v.4.6.8* `https://CRAN.R-project.org/package=wavethresh`. 2010.
16. Page, A. J. *et al.* SNP-sites: Rapid efficient extraction of SNPs from multi-FASTA alignments. *Microbial Genomics* **2** (2016).
17. Sagulenko, P., Puller, V. & Neher, R. A. TreeTime: Maximum-likelihood phylodynamic analysis. *Virus Evolution* **4,** vex042 (2018).
18. Shen, W., Le, S., Li, Y. & Hu, F. SeqKit: A cross-platform and ultrafast toolkit for FASTA/Q file manipulation. *PLOS ONE* **11,** e0163962 (2016).

19. Simmonds, P. Rampant C→U hypermutation in the genomes of SARS-CoV-2 and other coronaviruses: Causes and consequences for their short- and long-term evolutionary trajectories. *mSphere* **5** (2020).
20. van Dorp, L. *et al.* Emergence of genomic diversity and recurrent mutations in SARS-CoV-2. *Infection, Genetics and Evolution* **83,** 104351 (2020).
21. Wu, F. *et al.* A new coronavirus associated with human respiratory disease in China. *Nature* **579,** 265–269 (2020).

Other lineages			Lineage B.1.351		
Accession	Date	Ref	Accession	Date	Ref
EPI_ISL_660225	02/11/2020	SAO1	EPI_ISL_736958	20/11/2020	SAN1
EPI_ISL_660257	18/11/2020	SAO2	EPI_ISL_696481	19/11/2020	SAN2
EPI_ISL_736993	25/11/2020	SAO3	EPI_ISL_660637	03/11/2020	SAN3
EPI_ISL_660643	01/11/2020	SAO4	EPI_ISL_678632	11/11/2020	SAN4
EPI_ISL_660229	16/11/2020	SAO5	EPI_ISL_736932	25/11/2020	SAN5
EPI_ISL_736985	25/11/2020	SAO6	EPI_ISL_678641	12/11/2020	SAN6
EPI_ISL_736926	26/11/2020	SAO7	EPI_ISL_700422	04/11/2020	SAN7
EPI_ISL_696462	19/11/2020	SAO8	EPI_ISL_696503	25/11/2020	SAN8
EPI_ISL_660655	03/11/2020	SAO9	EPI_ISL_700470	12/11/2020	SAN9
EPI_ISL_660625	05/11/2020	SAO10	EPI_ISL_736983	24/11/2020	SAN10
EPI_ISL_660231	16/11/2020	SAO11	EPI_ISL_736936	19/11/2020	SAN11
EPI_ISL_678608	15/11/2020	SAO12	EPI_ISL_700487	06/11/2020	SAN12
EPI_ISL_660163	05/11/2020	SAO13	EPI_ISL_736935	26/11/2020	SAN13
EPI_ISL_660232	17/11/2020	SAO14	EPI_ISL_700443	13/11/2020	SAN14
EPI_ISL_700488	05/11/2020	SAO15	EPI_ISL_736939	24/11/2020	SAN15
EPI_ISL_660652	01/11/2020	SAO16	EPI_ISL_700554	02/11/2020	SAN16
EPI_ISL_660622	07/11/2020	SAO17	EPI_ISL_696505	25/11/2020	SAN17
EPI_ISL_660651	02/11/2020	SAO18	EPI_ISL_696518	24/11/2020	SAN18
EPI_ISL_678612	15/11/2020	SAO19	EPI_ISL_700589	12/11/2020	SAN19
EPI_ISL_696509	24/11/2020	SAO20	EPI_ISL_736959	20/11/2020	SAN20
EPI_ISL_678595	18/11/2020	SAO21	EPI_ISL_696453	20/11/2020	SAN21
EPI_ISL_660222	09/11/2020	SAO22	EPI_ISL_696521	24/11/2020	SAN22
EPI_ISL_696468	18/11/2020	SAO23	EPI_ISL_736964	19/11/2020	SAN23
EPI_ISL_660230	16/11/2020	SAO24	EPI_ISL_736928	24/11/2020	SAN24
EPI_ISL_660626	07/11/2020	SAO25	EPI_ISL_678629	13/11/2020	SAN25

Table S1. GISAID accession numbers, collection dates, and references of sequences in the South Africa (November) sample.

Accession	Date	Accession	Date
EPI_ISL_1048548	01/02/2021	EPI_ISL_1371925	15/02/2021
EPI_ISL_1048553	02/02/2021	EPI_ISL_1371926	09/02/2021
EPI_ISL_1048554	02/02/2021	EPI_ISL_1371927	19/02/2021
EPI_ISL_1048555	02/02/2021	EPI_ISL_1371928	21/02/2021
EPI_ISL_1048562	01/02/2021	EPI_ISL_1371929	20/02/2021
EPI_ISL_1366778	02/02/2021	EPI_ISL_1371930	09/02/2021
EPI_ISL_1366779	02/02/2021	EPI_ISL_1371931	21/02/2021
EPI_ISL_1366781	01/02/2021	EPI_ISL_1371932	17/02/2021
EPI_ISL_1366782	18/02/2021	EPI_ISL_1371933	23/02/2021
EPI_ISL_1366783	25/02/2021	EPI_ISL_1371995	05/02/2021
EPI_ISL_1366793	04/02/2021	EPI_ISL_1371996	15/02/2021
EPI_ISL_1366840	05/02/2021	EPI_ISL_1371999	09/02/2021
EPI_ISL_1366864	05/02/2021	EPI_ISL_1372000	07/02/2021
EPI_ISL_1366869	05/02/2021	EPI_ISL_1372001	08/02/2021
EPI_ISL_1366877	05/02/2021	EPI_ISL_1372002	09/02/2021
EPI_ISL_1366887	06/02/2021	EPI_ISL_1372003	24/02/2021
EPI_ISL_1366888	05/02/2021	EPI_ISL_1372004	18/02/2021
EPI_ISL_1371923	23/02/2021	EPI_ISL_1372005	17/02/2021
EPI_ISL_1371924	21/02/2021	EPI_ISL_1372006	17/02/2021

Table S2. GISAID accession numbers, collection dates, and references of sequences in the South Africa (February) sample.

Other lineages			Lineage B.1.1.7		
Accession	Date	Ref	Accession	Date	Ref
EPI_ISL_662468	12/11/2020	EO1	EPI_ISL_708881	30/11/2020	EN1
EPI_ISL_664402	06/11/2020	EO2	EPI_ISL_705071	22/11/2020	EN2
EPI_ISL_702752	19/11/2020	EO3	EPI_ISL_657548	09/11/2020	EN3
EPI_ISL_650455	13/11/2020	EO4	EPI_ISL_702338	27/11/2020	EN4
EPI_ISL_667977	14/11/2020	EO5	EPI_ISL_656730	08/11/2020	EN5
EPI_ISL_642566	02/11/2020	EO6	EPI_ISL_709730	26/11/2020	EN6
EPI_ISL_661404	11/11/2020	EO7	EPI_ISL_702093	28/11/2020	EN7
EPI_ISL_679726	01/11/2020	EO8	EPI_ISL_675080	15/11/2020	EN8
EPI_ISL_654967	10/11/2020	EO9	EPI_ISL_673518	15/11/2020	EN9
EPI_ISL_659205	05/11/2020	EO10	EPI_ISL_704716	30/11/2020	EN10
EPI_ISL_659013	01/11/2020	EO11	EPI_ISL_676036	13/11/2020	EN11
EPI_ISL_662253	11/11/2020	EO12	EPI_ISL_704695	02/11/2020	EN12
EPI_ISL_660027	04/11/2020	EO13	EPI_ISL_704619	21/11/2020	EN13
EPI_ISL_646293	04/11/2020	EO14	EPI_ISL_658341	08/11/2020	EN14
EPI_ISL_664758	12/11/2020	EO15	EPI_ISL_661750	14/11/2020	EN15
EPI_ISL_659140	05/11/2020	EO16	EPI_ISL_665414	02/11/2020	EN16
EPI_ISL_661929	14/11/2020	EO17	EPI_ISL_703736	26/11/2020	EN17
EPI_ISL_641906	03/11/2020	EO18	EPI_ISL_658292	08/11/2020	EN18
EPI_ISL_661483	11/11/2020	EO19	EPI_ISL_709568	26/11/2020	EN19
EPI_ISL_656165	06/11/2020	EO20	EPI_ISL_704601	22/11/2020	EN20
EPI_ISL_658415	08/11/2020	EO21	EPI_ISL_656409	08/11/2020	EN21
EPI_ISL_655916	08/11/2020	EO22	EPI_ISL_668252	12/11/2020	EN22
EPI_ISL_637180	02/11/2020	EO23	EPI_ISL_661854	12/11/2020	EN23
EPI_ISL_673482	15/11/2020	EO24	EPI_ISL_703229	19/11/2020	EN24
EPI_ISL_703087	19/11/2020	EO25	EPI_ISL_657799	08/11/2020	EN25
EPI_ISL_675115	13/11/2020	EO26	EPI_ISL_708945	30/11/2020	EN26
EPI_ISL_664943	04/11/2020	EO27	EPI_ISL_679428	22/11/2020	EN27
EPI_ISL_706068	02/11/2020	EO28	EPI_ISL_676194	13/11/2020	EN28
EPI_ISL_657282	08/11/2020	EO29	EPI_ISL_683471	24/11/2020	EN29
EPI_ISL_679916	06/11/2020	EO30	EPI_ISL_676012	13/11/2020	EN30
EPI_ISL_673815	15/11/2020	EO31	EPI_ISL_705063	22/11/2020	EN31
EPI_ISL_678719	16/11/2020	EO32	EPI_ISL_659491	05/11/2020	EN32
EPI_ISL_705061	19/11/2020	EO33	EPI_ISL_668018	12/11/2020	EN33
EPI_ISL_646457	03/11/2020	EO34	EPI_ISL_702918	19/11/2020	EN34
EPI_ISL_656970	08/11/2020	EO35	EPI_ISL_657622	08/11/2020	EN35
EPI_ISL_647347	01/11/2020	EO36	EPI_ISL_704698	01/11/2020	EN36
EPI_ISL_650406	08/11/2020	EO37	EPI_ISL_679302	21/11/2020	EN37
EPI_ISL_661700	13/11/2020	EO38	EPI_ISL_704606	22/11/2020	EN38
EPI_ISL_658474	08/11/2020	EO39	EPI_ISL_703148	19/11/2020	EN39
EPI_ISL_700654	09/11/2020	EO40	EPI_ISL_645527	05/11/2020	EN40

Table S3. GISAID accession numbers, collection dates, and references of sequences in the England (November) sample.

Accession	Date	Ref	Accession	Date	Ref
EPI_ISL_878756	13/01/2021	E1	EPI_ISL_868555	18/01/2021	E21
EPI_ISL_778191	20/12/2020	E2	EPI_ISL_885546	18/01/2021	E22
EPI_ISL_836766	04/01/2021	E3	EPI_ISL_816845	31/12/2020	E23
EPI_ISL_720681	02/12/2020	E4	EPI_ISL_736552	11/12/2020	E24
EPI_ISL_735634	13/12/2020	E5	EPI_ISL_731132	10/12/2020	E25
EPI_ISL_816235	29/12/2020	E6	EPI_ISL_820022	25/12/2020	E26
EPI_ISL_799427	22/12/2020	E7	EPI_ISL_1054040	30/01/2021	E27
EPI_ISL_777127	17/12/2020	E8	EPI_ISL_881303	12/01/2021	E28
EPI_ISL_811454	01/01/2021	E9	EPI_ISL_838888	04/01/2021	E29
EPI_ISL_1242096	27/01/2021	E10	EPI_ISL_950899	27/12/2020	E30
EPI_ISL_735656	13/12/2020	E11	EPI_ISL_709038	04/12/2020	E31
EPI_ISL_863458	13/01/2021	E12	EPI_ISL_842015	01/01/2021	E32
EPI_ISL_1178212	25/01/2021	E13	EPI_ISL_835329	05/01/2021	E33
EPI_ISL_777970	18/12/2020	E14	EPI_ISL_741276	08/12/2020	E34
EPI_ISL_782374	26/12/2020	E15	EPI_ISL_813970	26/12/2020	E35
EPI_ISL_868478	07/01/2021	E16	EPI_ISL_1051452	22/01/2021	E36
EPI_ISL_762877	16/12/2020	E17	EPI_ISL_1046024	27/01/2021	E37
EPI_ISL_1050650	29/01/2021	E18	EPI_ISL_836823	04/01/2021	E38
EPI_ISL_708906	04/12/2020	E19	EPI_ISL_994038	12/01/2021	E39
EPI_ISL_740955	02/12/2020	E20	EPI_ISL_820233	14/12/2020	E40

Table S4. GISAID accession numbers, collection dates, and references of sequences in the England (January) sample.

Accession	Submitters	Date	Ref
KY688118.1	Paden, C. R., et al.	07/02/2015	M1
KT806044.1	Lu, X., et al.	09/02/2015	M2
KT806045.1	Lu, X., et al.	22/02/2015	M3
KT806047.1	Lu, X., et al.	27/03/2015	M4
KT806048.1	Lu, X., et al.	07/02/2015	M5
KT806049.1	Lu, X., et al.	15/02/2015	M6
KT806051.1	Lu, X., et al.	05/02/2015	M7
KT806052.1	Lu, X., et al.	02/02/2015	M8
KT806053.1	Lu, X., et al.	02/02/2015	M9
KT806054.1	Lu, X., et al.	13/02/2015	M10
KT806055.1	Lu, X., et al.	10/02/2015	M11
KT026453.1	Park, W. B., et al.	10/02/2015	M12
KT026454.1	Park, W. B., et al.	01/03/2015	M13
KT026455.1	Park, W. B., et al.	10/02/2015	M14
KT026456.1	Park, W. B., et al.	01/03/2015	M15
KR011263.1	Lu, X., et al.	21/01/2015	M16
KR011264.1	Lu, X., et al.	21/01/2015	M17
KR011265.1	Lu, X., et al.	26/01/2015	M18
KR011266.1	Lu, X., et al.	06/01/2015	M19

Table S5. NCBI Virus database accession numbers, collection dates, and references of sequences in the MERS-CoV sample.

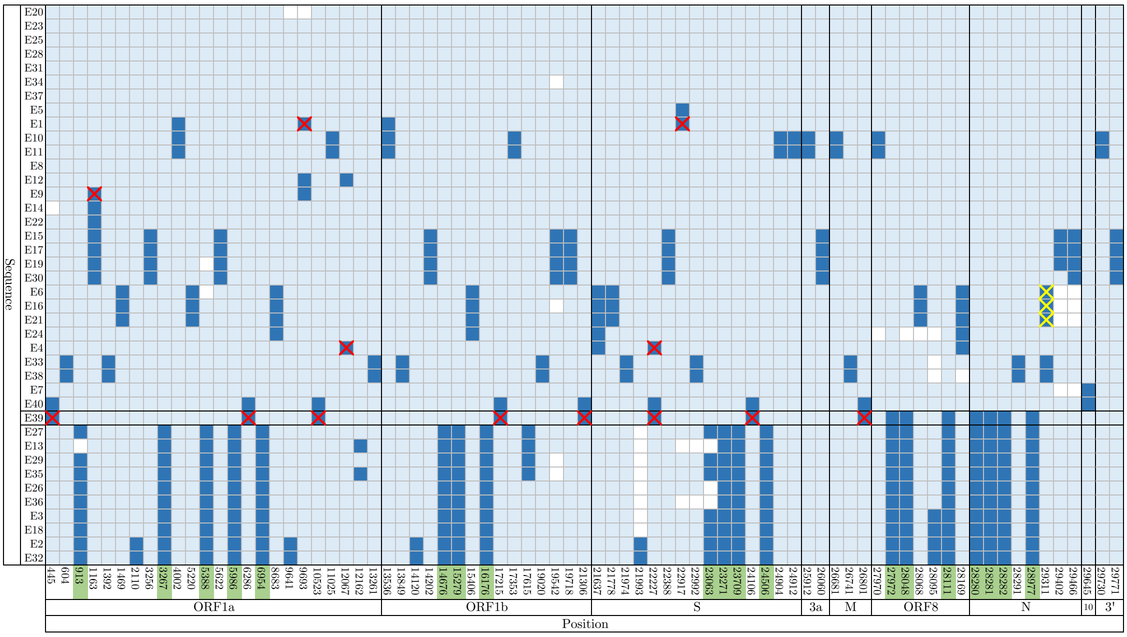

Figure S6. Summary of the England (January) dataset. Rows correspond to sequences, labelled on the left. Columns correspond to positions along the genome; uninformative sites (with all 0's or 1's) and those with singleton mutations (with exactly one 1) are not shown. Light blue: ancestral state, dark blue: mutated state, white: missing data. Red crosses highlight sites of recurrent mutations identified by KwARG located on the terminal branches of the ARG (affecting only one sequence). Yellow crosses highlight recurrent mutations on internal branches (hence affecting multiple sequences). Sites bearing the characteristic mutations of lineage B.1.1.7 (Rambaut *et al.*, 2020) are highlighted in green.

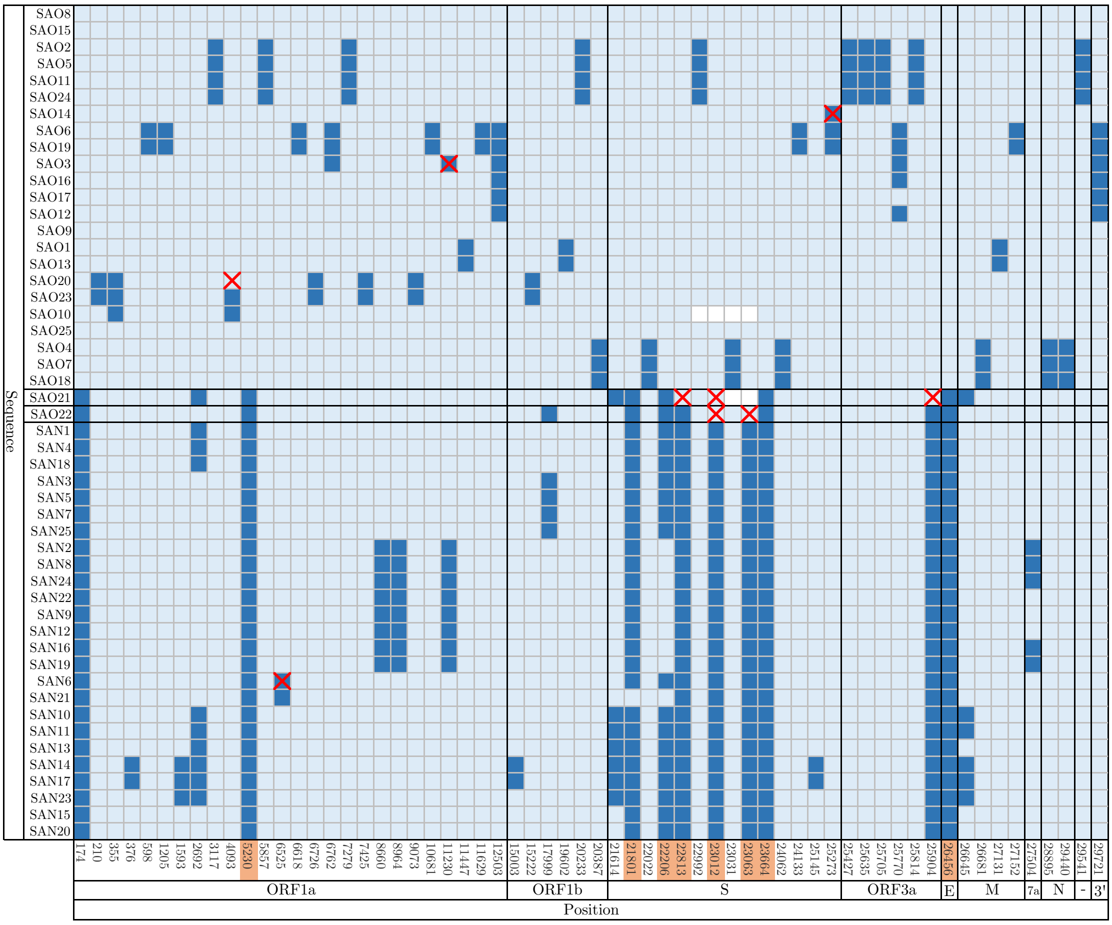

Figure S7. Summary of the South Africa (November) dataset. Rows correspond to sequences, labelled on the left. Columns correspond to positions along the genome; uninformative sites (with all 0's or 1's) and those with singleton mutations (with exactly one 1) are not shown. Light blue: ancestral state, dark blue: mutated state, white: missing data. Red crosses highlight sites of recurrent mutations identified by KwARG. Sites bearing the characteristic (non-synonymous) mutations of lineage B.1.351 (Tegally *et al.*, 2020) are highlighted in orange.

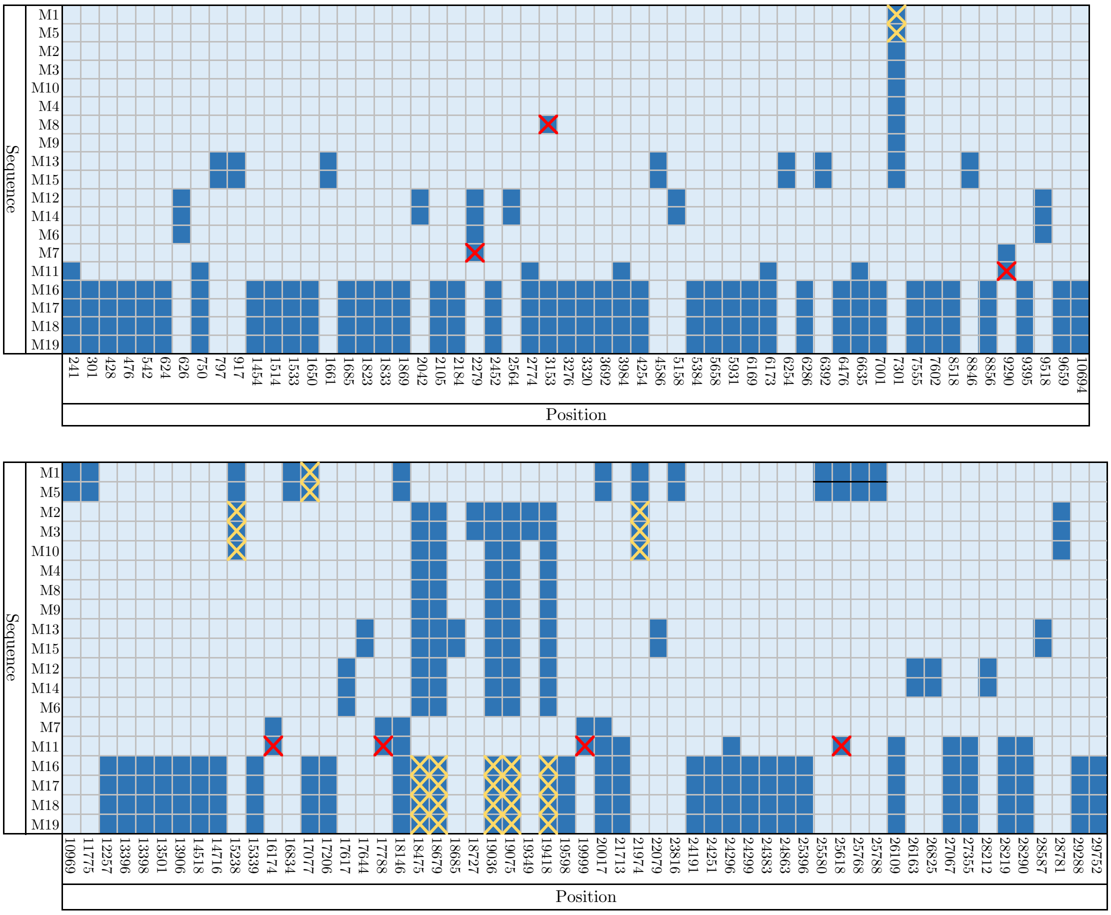

Figure S8. Summary of the MERS dataset. Rows correspond to sequences, labelled on the left. Columns correspond to positions along the genome; uninformative sites (with all 0's or 1's) and those with singleton mutations (with exactly one 0 or 1) are not shown. Light blue and dark blue denote differing allele types. Red crosses highlight sites of recurrent mutations identified by KwARG located on the terminal branches of the ARG (affecting only one sequence). Yellow crosses highlight recurrent mutations on internal branches (hence affecting multiple sequences).

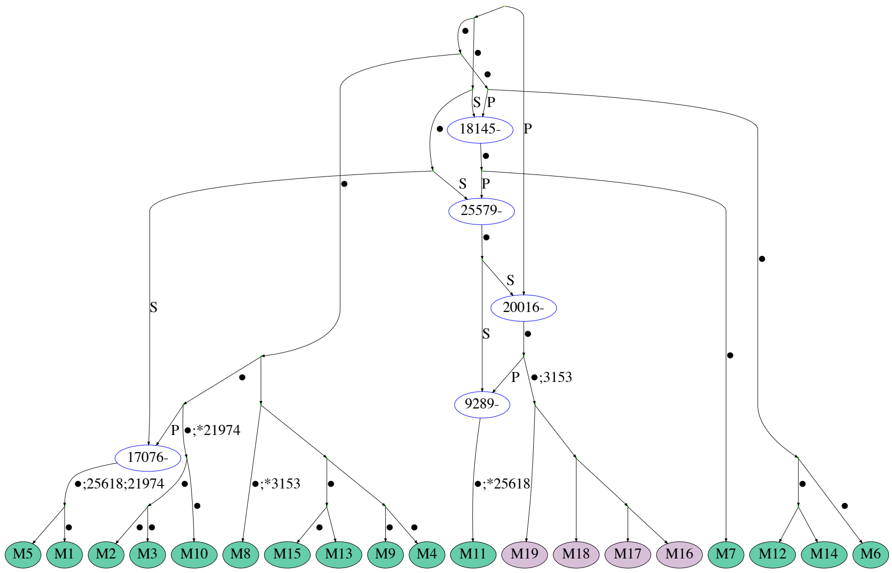

Figure S9. Example of an ARG for the MERS-CoV dataset. Recombination nodes are shown in blue, labelled with the recombination breakpoint, with the offspring sequence inheriting part of the genome to the left (right) of the breakpoint from the parent labelled “P” (“S”). Recurrent mutations are prefixed with an asterisk. Edges are labelled by positions of mutations (some mutated sites are not explicitly labelled and are denoted by a dot instead).
